# Cullin 7 mediates proteasomal and lysosomal degradations of rat Eag1 potassium channels

**DOI:** 10.1038/srep40825

**Published:** 2017-01-18

**Authors:** Po-Hao Hsu, Yu-Ting Ma, Ya-Ching Fang, Jing-Jia Huang, Yu-Ling Gan, Pei-Tzu Chang, Guey-Mei Jow, Chih-Yung Tang, Chung-Jiuan Jeng

**Affiliations:** 1 Institute of Anatomy and Cell Biology, School of Medicine, National Yang-Ming University, Taipei, Taiwan; 2 Department of Physiology, College of Medicine, National Taiwan University, Taipei, Taiwan; 3 Brain Research Center, National Yang-Ming University, Taipei, Taiwan; 4 School of Medicine, Fu-Jen Catholic University, Hsin-Chuang, New Taipei City, Taiwan

## Abstract

Mammalian Eag1 (Kv10.1) potassium (K^+^) channels are widely expressed in the brain. Several mutations in the gene encoding human Eag1 K^+^ channel have been associated with congenital neurodevelopmental anomalies. Currently very little is known about the molecules mediating protein synthesis and degradation of Eag1 channels. Herein we aim to ascertain the protein degradation mechanism of rat Eag1 (rEag1). We identified cullin 7 (Cul7), a member of the cullin-based E3 ubiquitin ligase family, as a novel rEag1 binding partner. Immunoprecipitation analyses confirmed the interaction between Cul7 and rEag1 in heterologous cells and neuronal tissues. Cul7 and rEag1 also exhibited significant co-localization at synaptic regions in neurons. Over-expression of Cul7 led to reduced protein level, enhanced ubiquitination, accelerated protein turn-over, and decreased current density of rEag1 channels. We provided further biochemical and morphological evidence suggesting that Cul7 targeted endoplasmic reticulum (ER)- and plasma membrane-localized rEag1 to the proteasome and the lysosome, respectively, for protein degradation. Cul7 also contributed to protein degradation of a disease-associated rEag1 mutant. Together, these results indicate that Cul7 mediates both proteasomal and lysosomal degradations of rEag1. Our findings provide a novel insight to the mechanisms underlying ER and peripheral protein quality controls of Eag1 channels.

The *ether-à-go-go* (Eag) K^+^ channel, belonging to the superfamily of voltage-gated K^+^ (K_V_) channels, is the founding member of the EAG family that comprises three gene subfamilies: *eag* (K_V_10), *erg (eag*-related gene, K_V_11), and *elk (eag*-like K^+^ channel, K_V_12)^1^. In mammals, there are two Eag isoforms: Eag1 (K_V_10.1) and Eag2 (K_V_10.2); both isoforms are widely expressed in many areas of the brain and exhibit differential subcellular localization in axonal and dendrosomatic compartments^2^,^3^,^4^,^5^,^6^,^7^,^8^,^9^,^10^,^11^,^12^,^13^. In *Drosophila*, where Eag1 K^+^ channels were initially identified, mutations in the *eag* gene result in distinct defects including hyper-excitability of neuromuscular junction and failure in learning courtship conditioning behavior^14^,^15^, suggesting an important role of Eag1 channels in synaptic transmissions. Consistent with this notion, mammalian Eag1, but not Eag2, is significantly expressed within the synaptic region and displays a distinct punctate localization pattern^10^,^12^,^13^.

Protein homeostasis (proteostasis) is regulated by a dynamic equilibrium between synthesis and degradation^16^,^17^,^18^. During the biosynthesis of ion channels, newly synthesized nascent proteins undergo molecular chaperone-guided folding, core-glycosylation, and subunit assembly in the endoplasmic reticulum (ER); properly folded and assembled channel proteins are then transported to the Golgi apparatus where they become full-glycosylated, followed by trafficking to the plasma membrane^19^,^20^. Upon failing to pass the inspection of the ER quality control system, misfolded or damaged channel proteins in the ER are marked with ubiquitins by E3 ubiquitin ligases and degraded at the proteasome; misfolded or damaged plasma membrane-resident channel proteins are also subject to ubiquitination by the peripheral quality control system, followed by a series of endosomal delivery events that eventually lead to the lysosome for degradation^20^,^21^,^22^. Both the proteasomal and the lysosomal degradation systems are tightly regulated to ensure timely removal of aberrant proteins in a highly selective manner^23^,^24^. The presence of elaborate protein degradation systems is essential for the maintenance of normal cellular functions, and emerging evidence supports the view that imbalances between the quality control system and the degradation machinery of ion channels can result in many human diseases^18^,^25^,^26^.

Very little is known about the molecular regulation of the synthesis and degradation of Eag K^+^ channels. To further address the potential physiological and pathological roles of Eag channels in the mammalian brain, herein we aim to ascertain the protein degradation mechanism of rat Eag1 (rEag1) proteins. We have identified cullin 7 (Cul7), a member of the cullin-based E3 ubiquitin ligase family^27^,^28^, as a potential binding partner of rEag1. By employing biochemical, morphological, and electrophysiological methods to characterize this novel protein interaction between Cul7 and rEag1, we conclude that Cul7 plays an important role in the molecular machinery dictating the ubiquitin-dependent regulation of rEag1 K^+^ channels in both the ER and the plasma membrane.

## Results

### Cul7 is a novel binding partner of rEag1

In order to search for rEag1-interacting proteins that may be involved in the regulation of protein ubiquitination and degradation, we carried out yeast two-hybrid screening of a rat brain cDNA library by using a bait comprising a cytoplasmic carboxyl-terminal region (amino acids 493–962) of rEag1 (see Suppl. Methods for more details) (Suppl. Fig. S1A). One of the positive clones isolated by the screening was Cul7. Cul7 serves as the scaffold protein that brings together the adaptor protein Skp1, the substrate-targeting subunit Fbw8, and the really interesting (RING)-domain protein ROC1 to form a cullin RING E3 ubiquitin ligase complex^27^,^28^.

**Figure 1:**
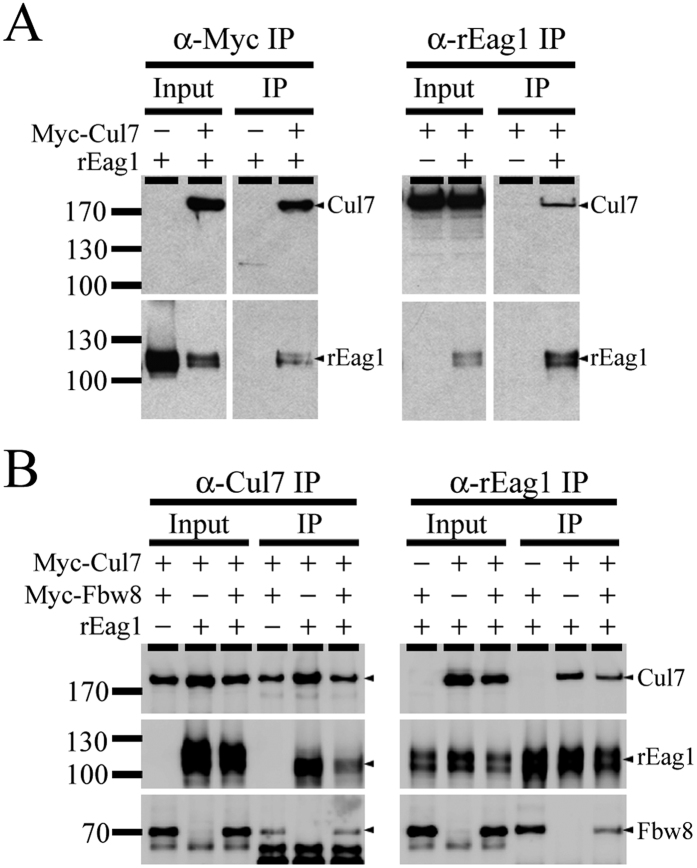
Interaction of rEag1 with Cul7 and Fbw8 in HEK293T cells. Representative immunoprecipitation data. (**A**) rEag1 was co-expressed with Myc-Cul7 or an empty vector in HEK293T cells. Cell lysates were immunoprecipitated (IP) by using the anti-Myc (α-Myc) or the anti-rEag1 (α-rEag1) antibodies, followed by immunoblotting analyses. Corresponding expression levels of rEag1 and Myc-Cul7 in the lysates are shown in the *Input* lane. Hereinafter, input volumes correspond to 5% of the total cell lysates used for immunoprecipitation. The positions of molecular mass markers (in kiloDalton, kDa) are indicated to the left. (**B**) Myc-Fbw8 was co-expressed with Myc-Cul7 or rEag1 in HEK293T cells, followed by immunoprecipitation and immunoblotting with the indicated antibodies. The protein bands corresponding to Cul7, rEag1, or Fbw8 are highlighted with arrowheads to the right. The gels were run under the same experimental conditions. Uncropped images of immunoblots are shown in Supplementary Figure S8.

To further confirm the interaction between Cul7 and rEag1, we performed immunoprecipitation experiments in HEK293T cells. Figure 1A illustrates that co-immunoprecipitation with rEag1 was detected in precipitates prepared from cells over-expressing rEag1 and Myc-tagged Cul7, but not from those over-expressing rEag1 and the vector control. In addition, GST pull-down assays identify rEag1 carboxyl-terminal CNBHD region as a key Cul7-interacting domain (Suppl. Fig. S1B-C). Our immunoprecipitation analyses further showed that the substrate receptor Fbw8 was co-immunoprecipitated with Cul7 (Fig. 1B, *left panel*), as well as with rEag1 (Fig. 1B, *right panel*). Altogether these observations suggest that rEag1 indeed co-exists in the same protein complex with the Cul7 E3 ubiquitin ligase.

### Interaction and co-localization of Cul7 with rEag1 in neurons

**Figure 2:**
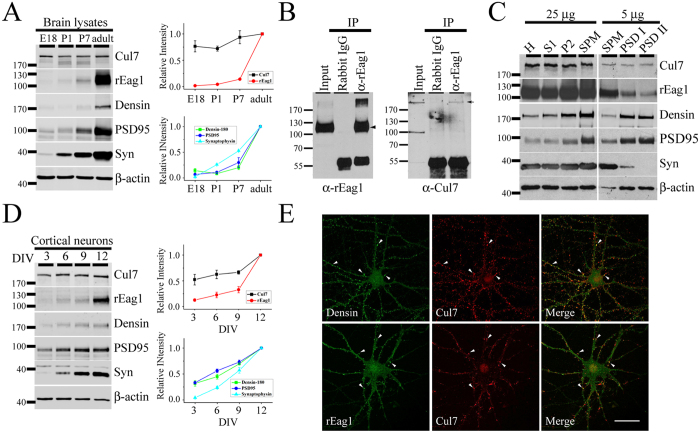
Association of rEag1 with Cul7 in neurons. (**A**) (*Left*) Developmental protein expression patterns of Cul7, rEag1, densin-180, PSD95, synaptophysin (Syn), and β-actin in brain lysates prepared from embryonic day 18 (E18), postnatal days 1 and 7 (P1, P7), and adult rats. (*Right*) Densitometric quantification of protein signals was standardized as the ratio to the cognate β-actin signals, followed by normalization to the adult group. Data were compiled from 3–5 independent experiments. (**B**) Co-immunoprecipitation of endogenous rEag1 and Cul7 in the brain. Rat forebrain lysates were immunoprecipitated with the anti-rEag1 antibody or the rabbit IgG, followed by immunoblotting with the indicated antibodies. The protein bands corresponding to rEag1 and Cul7 are highlighted with arrowhead and arrow, respectively. (**C**) Co-localization of rEag1 and Cul7 at pre- and post-synaptic compartments. Subcellular fractionation separated rat brains into homogenate (H), soluble (S1), crude membrane (P2), synaptosomal (SPM), and two postsynaptic density (PSD I: one Triton X-100 wash; PSD II: two Triton X-100 washes) fractions, all of which were subject to immunoblotting analyses with the indicated antibodies. 25 μg and 5 μg refer to the amount of total protein loaded in each lane. (**D**) (*Left*) Developmental protein expression patterns of Cul7, rEag1, densin-180, PSD95, synaptophysin, and β-actin in cultured hippocampal neurons with different days *in vitro* (DIV). (*Right*) Densitometric quantification of protein signals was standardized as the ratio to the cognate β-actin signals, followed by normalization to the DIV12 group. Data were compiled from 3 independent experiments. (**E**) Representative immunofluorescence images of rEag1, Cul7, and densin-180 in DIV12 hippocampal neurons. (*Upper panels*) Confocal micrographs showing the distribution of densin-180 (green) and Cul7 (red) puncta (arrowheads) along neurites and somas. (*Lower panels*) Confocal micrographs showing the co-localization of rEag1 (green) and Cul7 (red) puncta (arrowheads) along neurites. Co-localization of red and green puncta (yellow) is indicated in the merge images (see Suppl. Methods and Suppl. Fig. S2A for further quantitative analyses). These studies were reproduced in 3 independent experiments. Scale bar: 25 μm. The gels were run under the same experimental conditions. Uncropped images of immunoblots are shown in Supplementary Figure S8.

Given that both Cul7 and rEag1 are profusely expressed in the brain, it is crucial to determine whether the potential interaction of these two proteins can also be verified in neurons. We first examined the protein expression profile of endogenous rEag1 and Cul7 in brain lysates prepared from different developmental stages of rats. Figure 2A depicts that the expression of the synaptic proteins densin-180, PSD95, and synaptophysin visibly increased as the brain matured from embryonic day 18 (E18), postnatal days 1 and 7 (P1, P7), to adulthood. rEag1 protein level also displayed a dramatic increase from P7 to adulthood; in contrast, Cul7 expression was relatively stable, only showing a moderate growth from P1 to adulthood. Therefore, we performed immunoprecipitation experiments in crude membrane fractions prepared from forebrain homogenates of adult rats. As exemplified in Fig. 2B, Cul7 was co-immunoprecipitated with rEag1, suggesting that endogenous Cul7 and rEag1 also co-exist in the same protein complex in the brain.

Previous biochemical and immunofluorescence studies from our lab strongly suggest that rEag1 is present at both presynaptic and postsynatipc regions in neurons^10^,^12^. We therefore asked whether Cul7 and rEag1 shared overlapping subcellular localization in neurons by performing fractionation analyses, which separate adult rat forebrain homogenates into multiple subcellular fractions. As illustrated in Fig. 2C, both Cul7 and rEag1 proteins were present in the synaptosomal (SPM) fraction. Extractions with Triton X-100 further divided the SPM fraction into two postsynaptic density fractions (PSD I and PSD II): the postsynaptic proteins densin-180 and PSD95 were highly enriched in both the PSD I and the PSD II fractions, whereas only trace amount of the presynaptic marker synaptophysin was present in the PSD I fraction. Importantly, Cul7 as well as rEag1 were detected in both the PSD I and the PSD II fractions, suggesting that Cul7 and rEag1 co-exist in close vicinity at the synaptic region.

We also examined the subcellular localization of endogenous Cul7 and rEag1 in cultured hippocampal neurons prepared from rat E18 embryos. Figure 2D shows that cultured neurons matured with increasing day *in vitro* (DIV), as demonstrated by the progressive up-regulation of the synaptic proteins densin-180, PSD95, and synaptophysin. Moreover, both Cul7 and rEag1 were abundantly expressed in DIV12 neurons. We then carried out immunofluorescence characterizations of Cul7 and rEag1 in DIV12 neurons. Figure 2E and Supplementary Figure S2A illustrate that, like rEag1, Cul7 displayed a prominent punctate pattern along neuronal processes that notably co-localized with the synaptic protein densin-180, suggesting that Cul7 and rEag1 puncta share overlapping localization at the synaptic region. Collectively, the preceding results indicate that endogenous Cul7 and rEag1 exhibit significant co-localization in the brain.

### Cul7 regulates rEag1 protein level

**Figure 3:**
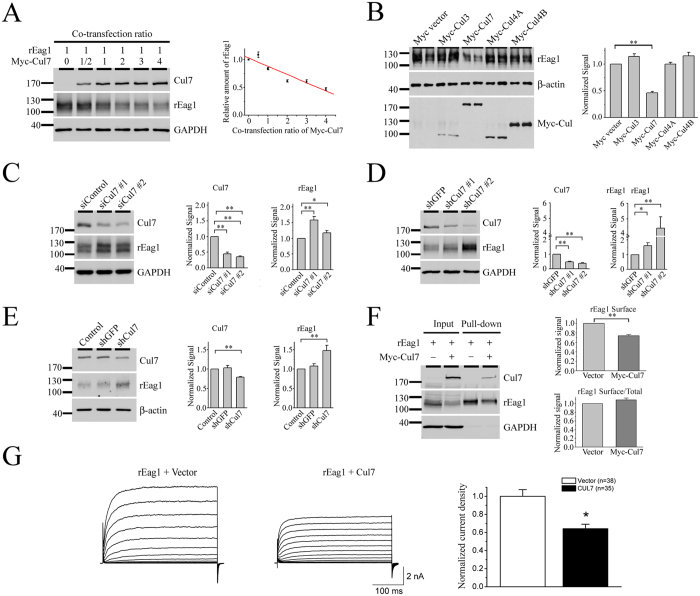
Regulation of rEag1 protein expression by Cul7. (**A**) Cul7 over-expression reduces rEag1 protein level in HEK293T cells. (*Left*) Representative rEag1 protein expression in response to increasing co-transfection ratios of Cul7. The expression of GAPDH is shown as the loading control. (*Right*) Quantification of relative rEag1 protein levels with respect to Cul7 co-transfection ratios. rEag1 signals were standardized as the ratio to the cognate GAPDH signals, followed by normalization to the no Cul7 control. Data were compiled from 3 independent experiments. (**B**) Co-expression of rEag1 with Myc-tagged Cullin 3, 4 A, 4B, or 7 in the molar ratio 1:2 in HEK293T cells. Compared to the Myc-vector control, only Cul7 displayed a significant (**p < 0.01; n = 4–6) repression effect on rEag1 protein expression. (**C**,**D**) siRNA or shRNA suppression of endogenous Cul7 expression enhances rEag1 protein level in HEK293T cells (*p < 0.05; **p < 0.01; n = 5). (**E**) shRNA-mediated silencing of endogenous Cul7 expression results in the up-regulation of endogenous rEag1 protein level in cultured hippocampal neurons (**p < 0.01; n = 3). β-actin signals were employed for quantification standardization. (**F**) Cul7 reduces surface expression of rEag1 in HEK293T cells (**p < 0.01; n = 5). Cells were biotinylated on the cell surface with Sulfo-NHS-LC-Biotin and soluble extracts were pulled down with streptavidin-agarose. (**G**) Cul7 reduces functional expression of rEag1 channels in HEK293T cells. (*Left*) Representative rEag1 K^+^ current traces in the absence or presence of Cul7 co-expression. The holding potential was −90 mV, and the pulse potentials ranged from −80 to +60 mV. (*Right*) Quantification of rEag1 whole-cell current density at +40 mV (*p < 0.05). The numbers of observations are shown in parentheses. The gels were run under the same experimental conditions. Uncropped images of immunoblots are shown in Supplementary Figure S8.

The next question we addressed was whether rEag1 is a potential substrate protein of the Cul7 E3 ubiquitin ligase. By co-expressing Cul7 with rEag1 in increasing molar ratios, we observed a significant concentration-dependent down-regulation of rEag1 by Cul7 (Fig. 3A); for example, with the Cul7 co-transfection ratios 1:2 and 1:4, rEag1 level was reduced by about 40% and 60%, respectively. Cullin E3 ligases constitute the largest subfamily of human E3 ubiquitin ligases, and human cells are known to express several different subtypes of cullins, each serving as the core of a subclass of cullin RING ubiquitin ligases^29^,^30^,^31^. Figure 3B demonstrates that, with a 1:2 co-transfection ratio, Cul7 significantly suppressed rEag1 protein expression; in contrast, no discernible effects were observed for Cul3, Cul4A, or Cul4B.

To further characterize the effect of Cul7 on rEag1, we also knocked down endogenous Cul7 expression in HEK293T cells using siRNA transfection or shRNA lentiviral infection. In both cases, Cul7 knock-down resulted in significant increase of rEag1 protein level (Fig. 3C and D). Consistent with these observations, rEag1 up-regulation was also detected when we applied MLN4924 (Suppl. Fig. S3A), which is a potent inhibitor of cullin RING E3 ligases^32^,^33^. Moreover, comparable results were obtained when we employed shRNA knock-down of endogenous Cul7 expression in rat B35 neuroblastoma cells (Suppl. Fig. S3B), suggesting that the foregoing RNA interference effects are not merely a special case for the HEK293T heterologous expression system. Importantly, shRNA knock-down of Cul7 led to notable enhancement of endogenous rEag1 level in cultured cortical neurons as well (Fig. 3E), thereby confirming the regulation of rEag1 level by Cul7 in neurons.

In addition to studying the effect on total protein level, we also employed cell surface biotinylation assays to demonstrate that surface rEag1 was significantly reduced in the presence of Cul7 over-expression (Fig. 3F). Furthermore, Fig. 3G and Supplementary Figure S4 show that co-expression with Cul7 in the molar ratio 1:2 led to substantial decrease of rEag1 whole-cell current density in HEK293T cells, consistent with the idea that the Cul7 E3 ubiquitin ligase also regulates the amount of rEag1 protein available at the plasma membrane.

### Cul 7 reduces protein stability of rEag1

**Figure 4:**
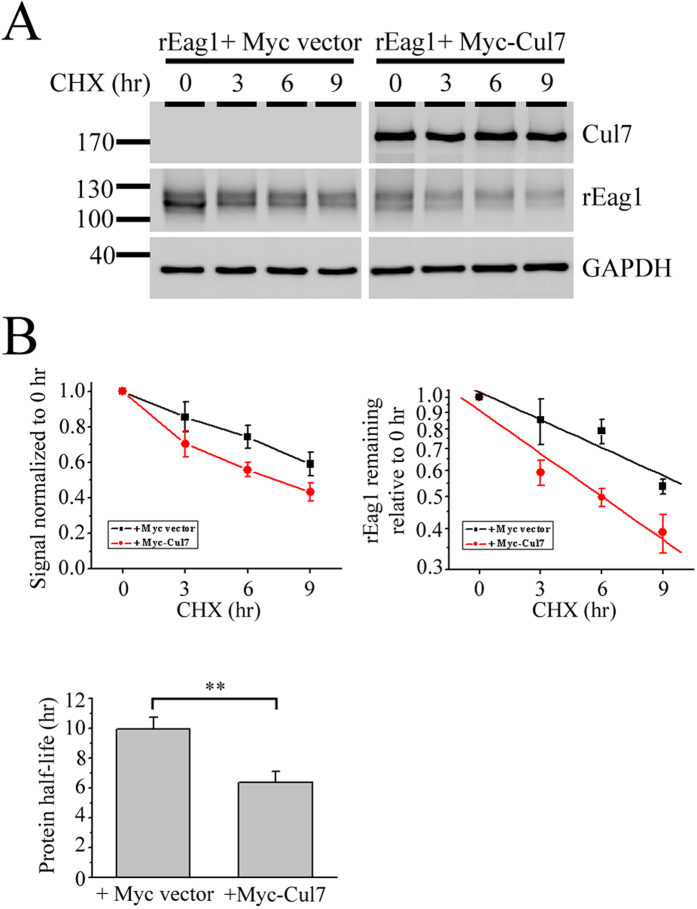
Regulation of rEag1 protein stability by Cul7. (**A**) Representative immunoblots showing the turn-over time course of total rEag1 protein in HEK293T cells in the absence or presence of Cul7. Transfected cells were subject to 100 μg/ml cycloheximide (CHX) treatment for the indicated durations. (**B**) Quantification of rEag1 protein degradation time course. (*Left*) rEag1 protein densities were standardized as the ratio of rEag1 signals to the cognate GAPDH signals, followed by normalization to those of the corresponding control at 0 hr. Data points represent the average of 5 independent experiments. (*Right*) Linear-regression analyses (*solid lines*) of the semi-logarithmic plot of the same data points shown to the left. (**C**) Statistical analyses of rEag1 protein half-life in the absence or presence of Cul7 (**p < 0.01; n = 5). The protein degradation time course determined from each experiment was individually plotted on a semi-logarithmic scale for linear-regression analysis. The gels were run under the same experimental conditions. Uncropped images of immunoblots are shown in Supplementary Figure S8.

In theory, Cul7 may down-regulate rEag1 protein level via reduced protein synthesis or enhanced protein degradation. Co-expression with Cul7 did not appear to detectably affect rEag1 mRNA level in HEK293T cells (data not shown). In contrast, by performing cycloheximide chase experiments, we found that Cul7 co-expression resulted in a significant acceleration of rEag1 protein turnover rate: the protein half-life of rEag1 was dramatically reduced by about 40% (from about 10 to about 6 hours) (Fig. 4). Together, these results confirm that Cul7 indeed down-regulates rEag1 protein level through enhanced protein degradation.

### Cul7 degrades rEag1 via both proteasomal and lysosomal pathways

**Figure 5:**
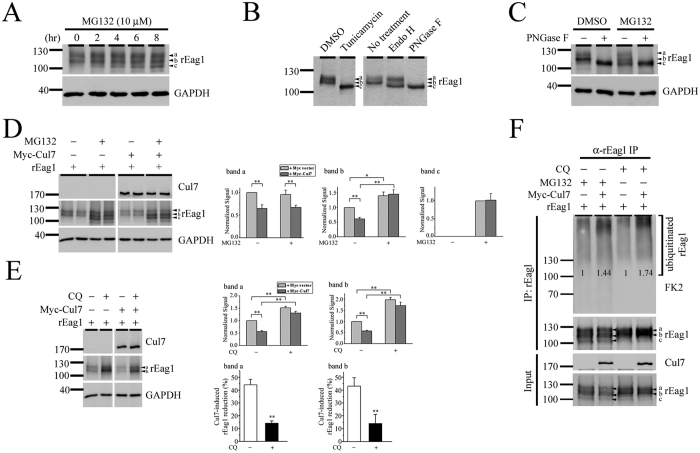
Cul7 degrades rEag1 via both proteasomal and lysosomal pathways. (**A**) Proteasome inhibition with 10 μM MG132 (in DMSO). In the absence of MG132, rEag1 expression in HEK293T cells is characterized by two protein bands (a, b) of 110–120 kDa. Increasing MG132 treatment durations leads to enhanced bands a and b signals, as well as the presence of an additional band (*c*) with the lowest molecular weight. (**B**) Glycan structure modification with tunicamycin, Endo H, or PNGase F exerts distinct effects on the three rEag1 protein bands. (**C**) PNGase F and MG132 treatments result in the appearance of apparently identical rEag1 protein band c. (**D**) MG132 treatment reverses Cul7 effect on rEag1 protein band b. (*Left*) Representative immunoblots showing Cul7 effects in the absence or presence of MG132 treatment. Each experimental result is displayed in duplicates. (*Right*) Quantification of Cul7 effects on the three rEag1 protein bands in the absence or presence of MG132 treatment (see Suppl. Fig. S5A for detailed differentiation of rEag1 bands) (*p < 0.05; **p < 0.01; n = 3). Protein densities for each rEag1 protein band were standardized as the ratio of rEag1 signals to the cognate GAPDH signals, followed by normalization to those of the corresponding vector/DMSO control. (**E**) Lysosome inhibition with 100 μM chloroquine (CQ) (in DMSO) reverses the effect of Cul7 on rEag1 protein bands a and b. (*Left*) Representative immunoblots showing Cul7 effects in the absence or presence of chloroquine treatment. (*Right*) Quantification of Cul7 effects on the three rEag1 protein bands in the absence or presence of chloroquine treatment (*p < 0.05; **p < 0.01; n = 7). (**F**) Cul7-mediated protein ubiquitination of rEag1 is enhanced by MG132 or chloroquine treatments. Ubiquitin was detected with the anti-ubiquitin antibody FK2. Ubiquitinated rEag1 was visualized as protein smears with high molecular weights. The numbers denote quantification of relative ubiquitinated rEag1 levels with respect to the corresponding vector control. The gels were run under the same experimental conditions. Uncropped images of immunoblots are shown in Supplementary Figure S8.

Since both total and surface rEag1 protein levels are reduced in the presence of Cul7, it is conceivable that the Cul7 ubiquitin ligase may target ER- and plasma membrane-localized rEag1 to the proteasome and the lysosome, respectively, for protein degradation. To address this hypothesis, we began by examining the effect of the specific proteasome inhibitor MG132 on rEag1. Immunoblotting analyses in Fig. 5A (see also Figs 1, 2, 3 and 4) depict that the electrophoretic mobility of rEag1 predominantly manifests two protein bands: a slower band “a” and a faster band “b”. However, suppression of proteasomal degradation with 10 μM MG132 led to a significant treatment duration-dependent accumulation of a third rEag1 protein band (band “c”) with the lowest apparent molecular weight. An important step during the biogenesis of membrane protein is the conversion of its glycosylation pattern from core (high mannose oligosaccharides) into mature (complex) glycan structures^34^. Therefore, it is likely that bands a, b, and c may represent different states of rEag1 glycosylation. Consistent with this notion, treatment with tunicamycin, an asparagine (N)**-**glycosylation inhibitor that virtually obliterates all N-link glycosylation, resulted in the collapse of bands a and b into a single band whose molecular weight resembles that of the MG132-induced band c (Fig. 5B). Treatment with N-glycosidase F (PNGase F), an amidase that removes all N-linked oligosaccharides from glycosylated proteins, also resulted in a similar low-molecular-weight band (Fig. 5B). The immunoblot in Fig. 5C further illustrates that the lowest-molecular-weight protein band in response to either PNGase F or MG132 exhibited no detectable difference in their apparent electrophoretic mobility, suggesting that an identical rEag1 band c is observed following MG132, tunicamycin, or PNGase F treatments. In contrast, treatment with endoglycosidase H (Endo H), which cleaves high mannose oligosaccharides from immature, core-glycosylated glycoproteins, led to the collapse of band b, but not band a, into band c (Fig. 5B). Taken together, these observations are consistent with the idea that band a may represent mature, full-glycosylated rEag1 localized at the plasma membrane, and that band b may correspond to immature, core-glycosylated rEag1 in the ER. On the other hand, band c is most likely equivalent to the nascent, non-glycosylated form of rEag1 proteins in the ER.

The aforementioned data in Figs 3 and 4 clearly demonstrate that Cul7 down-regulates both full- and core-glycosylated forms of rEag1 proteins. In contrast, since band c was not observed when we suppressed endogenous Cul7 expression/function with RNA interference or MLN4924 (see Fig. 3 and Suppl. Fig. S3A), we infer that Cul7 does not appear to significantly contribute to the degradation of non-glycosylated rEag1. Therefore, we moved on to investigate the effect of MG132 on Cul7-induced degradation of full- and core-glycosylated rEag1 proteins, *i.e.*, bands a and b. Figure 5D and Supplemental Figure S5A show that treatment with MG132 notably rescued Cul7-mediated down-regulation of the immature rEag1 band b, but not the mature rEag1 band a, suggesting that Cul7 only targets ER-localized, but not plasma membrane-localized, rEag1 for proteasomal degradation. Similar results were also observed when we repeated the same experiment with another proteasome inhibitor, ALLN (data not shown).

As mentioned in the Introduction section, damaged or misfolded plasma membrane-resident proteins are recognized by the peripheral quality control system and degraded by the endosome-lysosomal system. To determine whether Cul7 may target plasma membrane-localized rEag1 for lysosomal degradation, we treated transfected HEK293T cells with the lysosome inhibitor chloroquine. As depicted in Fig. 5E, in direct contrast with the effect of MG132, chloroquine did not appreciably induce the presence of rEag1 band c. Also unlike MG132, chloroquine significantly reversed the effect of Cul7 on rEag1 band a, in accordance with the notion that membrane-localized, full-glycosylated rEag1 is subject to lysosomal degradation. Finally, protein ubiquitination analyses provided further evidence showing that Cul7 enhanced both MG132- and chloroquine-sensitive ubiquitination of rEag1 proteins (Fig. 5F). In contrast, in the absence of MG132 and chloroquine, Cul7 co-expression failed to discernibly alter protein ubiquitination of rEag1 (Suppl. Fig. S5B). Altogether, these data are consistent with the idea that rEag1 is subject to significant Cul7-mediated ubiquitination prior to proteasomal or lysosomal degradations.

**Figure 6:**
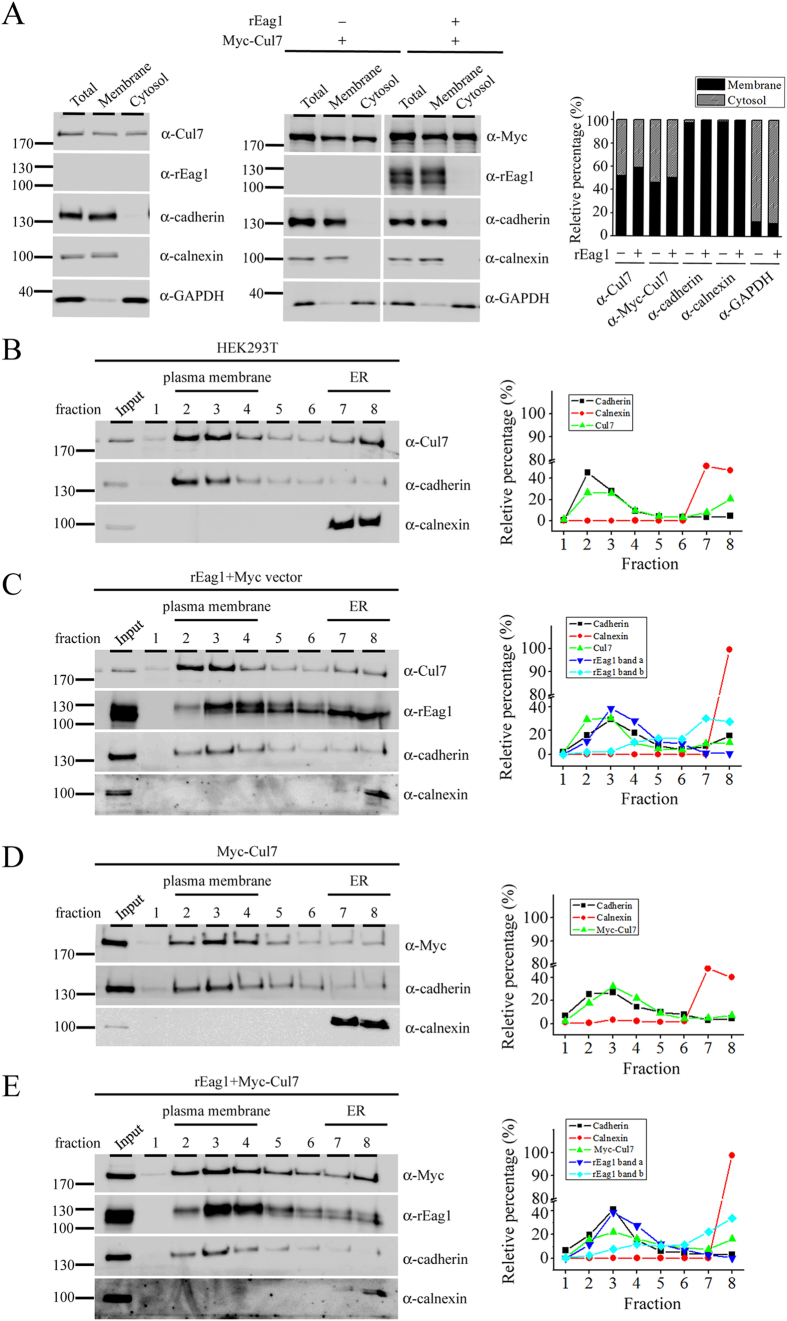
Centrifugation and fractionation analyses support the localization of Cul7 at the plasma membrane. HEK293T cell homogenates were analyzed by differential centrifugation (**A**) or sucrose gradient fractionation (**B**–**E**). In both cases, total homogenates (Total) were ultracentrifuged and thereafter separated into the supernatant (Cytosol) and the pellet (Membrane) fractions. Three endogenous proteins in HEK293T cells were used as specific markers for distinct subcellular compartments: cadherin (plasma membrane protein), calnexin (ER-resident membrane-associated protein), and GAPDH (cytosolic protein). (**A**) In differential centrifugation analyses, both endogenous Cul7 (detected by the anti-Cul7 antibody) and over-expressed Myc-Cul7 (detected by the anti-Myc antibody) were found to be present in the cytosol as well as the membrane fractions. In contrast, rEag1 was present in the membrane fraction only. Shown to the right is the quantification of the relative percentage of membrane and cytosol distributions (with respect to the total signal) for each protein (n = 3). (**B**–**E**) In sucrose gradient fractionation analyses, the membrane pellet fraction (Input) was sedimented through a discontinuous sucrose gradient and further divided into 8 fractions. Fractions 2–4, wherein cadherin was detected, correspond to the plasma membrane fractions. Fractions 7–8, wherein calnexin was detected, indicate the ER membrane fractions. Shown to the right is the densitometric quantification of each representative immunoblot that highlights the relative distribution (with respect to the total signal) in different fractions for a specific protein. (**B**) Endogenous Cul7 in HEK293T cells was predominantly found in the plasma membrane fractions, and to a lesser extent in the ER membrane fractions. (**C**) rEag1 protein bands a and b were preferentially detected in the plasma membrane and the ER membrane fractions, respectively. (**D**) A significant portion of over-expressed Myc-Cul7 was localized in the plasma membrane fractions. (**E**) Myc-Cul7 and rEag1 protein band a co-localized in the plasma membrane fractions. The gels were run under the same experimental conditions. Uncropped images of immunoblots are shown in Supplementary Figure S8.

Another line of evidence supporting a protein degradation role of Cul7 at both the ER and the plasma membrane concerns the subcellular localization of Cul7. Differentiation centrifugation analyses in HEK293T cells clearly show that endogenous Cul7, as well as over-expressed Myc-Cul7, was present in both the cytosol and the membrane fractions (Fig. 6A). Moreover, sucrose density gradient analyses suggest that endogenous Cul7 were found in the plasma membrane and the ER membrane fractions (Fig. 6B), and that rEag1 protein bands a and b were preferentially detected in the plasma membrane and the ER membrane fractions, respectively (Fig. 6C). Importantly, Fig. 6D,E further illustrate that a significant portion of Myc-Cul7 co-localized with rEag1 band a at the plasma membrane.

To further address the effect of Cul7 on surface rEag1, we also used brefeldin A (BFA) chase assays to investigate the effect of Cul7 co-expression on the degradation rate of plasma membrane-resident rEag1. BFA blocks the forward trafficking of proteins from the ER to the Golgi^35^, thereby preventing the conversion of rEag1 from the immature form (band b) into the mature form (band a). As a result of BFA-induced blockade of forward trafficking, the time course of band a reduction in the presence of BFA may directly reflect the turnover rate of mature rEag1 channels at the cell surface. Supplementary Figure S6 demonstrates that, in the presence of Cul7, the protein half-life of rEag1 band a visibly decreased by about 50% (from about 10.7 to about 6.8 hours), implying that Cul7-over-expression accelerates the degradation rate of surface rEag1.

**Figure 7:**
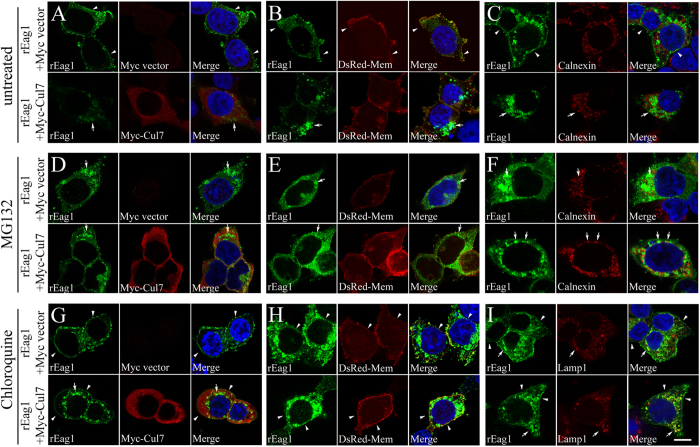
Pharmacological suppression of proteasomal and lysosomal degradations alters subcellular localization of rEag1 in HEK293T cells. Representative confocal micrographs showing rEag1 immunofluorescence signals (green) in response to Cul7 co-expression (**A**,**B**,**C**), or 6-hour MG132 (**D**,**E**,**F**) or chloroquine (**G**,**H**,**I**) treatment. rEag1 and Myc-Cul7 were detected with anti-rEag1 and anti-Myc antibodies, respectively. Nuclei were counterstained with DAPI (blue). To verify plasma membrane localization, some cells were co-transfected with the DsRed-membrane expression vector (DsRed-Mem). ER and lysosomal localizations were detected by specific antibodies for the ER marker calnexin and the lysosome marker lamp1, respectively. Merge images are shown in the third column of each panel. Arrowheads indicate plasma membrane staining, whereas arrows denote intracellular staining. See Supplementary Methods and Supplementary Figure S2B for further quantitative analyses. Scale bar, 10 μm. Data shown here are representative of over 80 cells from at least 3 independent experiments.

In addition, we studied the effect of Cul7 co-expression on the subcellular localization of rEag1 in HEK293T cells by employing confocal laser scanning microscopy. Figure 7A–C demonstrate that rEag1 immunofluoresence signal manifested a reticular perinuclear pattern, as well as a thin-line membrane pattern. Furthermore, Fig. 7B and Supplementary Figure S2B show that co-expression with Cul7 resulted in a reduction of membrane staining of rEag1, as indicated by the significantly decreased co-localization of rEag1 with the plasma membrane marker DsRed-membrane. These results suggest that Cul7 may induce a redistribution of membrane rEag1 to perinuclear localization in the cytoplasm. Figure 7D–F exemplify the effect of blocking proteasomal degradation with MG132 on the subcellular localization of rEag1. Interestingly, in the presence of MG132, Cul7 elicited a more notable co-localization of rEag1 with calnexin, an ER-resident protein (Fig. 7F; Suppl. Fig. S2B). Figure 7G–I highlight the effect of blocking lysosomal degradation with chloroquine on the subcellular localization of rEag1. Importantly, in the presence of chloroquine, Cul7 promoted the co-localization of rEag1 with the lysosomal marker lamp1 (Fig. 7I; Suppl. Fig. S2B). Overall, these observations seem to indicate that, in the presence of Cul7, pharmacological suppression of proteasomal and lysosomal degradations may render rEag1 more susceptible for ER and lysosome retentions, respectively.

### Cul7 contributes to disease-associated degradation of rEag1 protein

**Figure 8:**
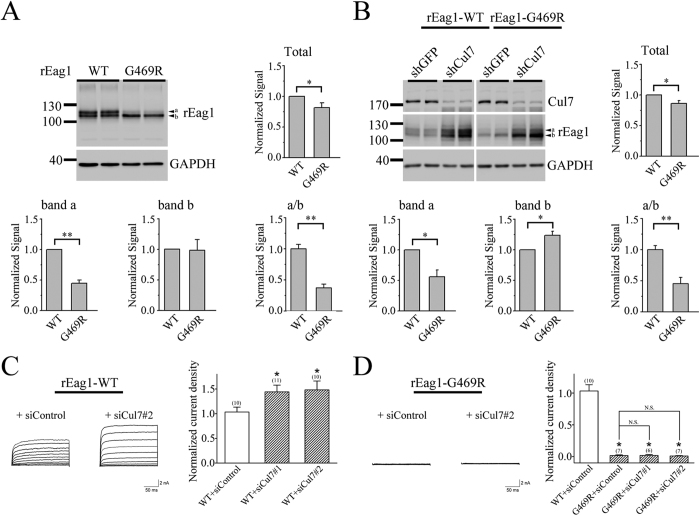
Reduced protein expression of the disease-associated rEag1-G469R mutant. (**A**) Representative immunoblot comparing protein expression of rEag1 wild-type (WT) and G469R mutant in HEK293T cells. Statistical comparisons between wild-type and G469R were performed for total protein level, protein bands a and b signals, as well as the relative signal ratio of band a to band b (a/b) (*p < 0.05; **p < 0.01; n = 3). (**B**) shRNA knock-down of endogenous Cul7 expression in HEK293T cells increases the relative protein level of G469R band b (*p < 0.05; **p < 0.01; n = 5). Each experimental result is displayed in duplicates. Protein densities in response to Cul7 knock-down were standardized as the ratio to the cognate GAPDH signals, followed by normalization to the wild-type. (**C**,**D**) The effect of siRNA knock-down of endogenous Cul7 expression on current expression of rEag1 WT and G469R mutant in HEK293T cells. (*Left*) Representative K^+^ current traces in the absence or presence of Cul7 siRNA knock-down. (*Right*) Quantification of rEag1 whole-cell current density at +40 mV (*p < 0.05; N.S., not significant). The numbers of observations are shown in parentheses. The gels were run under the same experimental conditions. Uncropped images of immunoblots are shown in Supplementary Figure S8.

In human, Eag1 K^+^ channel protein is encoded by the *KCNH1* gene. Several *KCNH1* gene mutations are associated with two congenital neurodevelopmental anomalies, the Temple-Baraitser syndrome (TMBTS) and the Zimmermann-Laband syndrome (ZLS)^36^,^37^,^38^. One of the ZLS-related mutations yields a single amino acid replacement at the S6 transmembrane segment (G469R) that manifests a complete loss of K^+^ channel function, a defect that was proposed to arise from a steric hindrance imposed by the arginine residue at the pore region of Eag1 channel^37^. However, an additional explanation for the loss-of-function phenotype of the G469R mutant is that the mutation instigates a serious disruption of Eag1 biosynthesis, thereby resulting in severe ER retention and/or enhanced protein degradation. To address the latter hypothesis, we introduced the same mutation into rEag1 channel, followed by biochemical characterizations in HEK293T cells. Figure 8A highlights that, compared to its wild-type (WT) counterpart, rEag1-G469R displayed a reduction in total protein level, which can be attributed to a significant decrease of band a, but not band b. To further determine whether Cul7 may contribute to the observed enhanced degradation of the mutant, we examined the effect of Cul7-specific shRNA infection on G469R protein expression. Figure 8B illustrates that, in response to Cul7 knock-down, the band b signal of G469R became significantly stronger than that of WT. Nevertheless, although the same treatment did visibly enhance the band a signal of G469R, shRNA knock-down of endogenous Cul7 in HEK293T cells fell short of rescuing the mutant’s deficit in total protein expression. Furthermore, siRNA knock-down of endogenous Cul7 expression substantially enhanced the current expression of rEag1 WT channels in HEK293T cells (Fig. 8C). In contrast, regardless of whether Cul7 expression was suppressed or not, no detectable currents were observed for the G469R mutant (Fig. 8D). Taken together, these results seem to suggest that the majority of the Cul7 shRNA/siRNA-rescued rEag1 mutant protein fails to pass the ER quality control system and thus remains trapped in the ER.

## Discussion

Cul7 is the structural backbone (scaffold protein) of the Cul7 E3 ubiquitin ligase complex, which includes three additional protein modules: the F-box protein Fbw8 that works as the substrate-binding protein, the adaptor protein Skp1 that connects the substrate receptor to the Cul7 scaffold, and the RING finger protein ROC1 (Rbx1) that contains the E2 enzyme-binding domain^27^,^30^. Not much is known about the role of Cul7 in neuronal cells. One of the identified substrate proteins of the Cul7 E3 ubiquitin ligase is a Golgi apparatus reassembly-stacking protein (GRASP65) that controls Golgi morphology and dendrite elaboration during the development of cerebellar granule cells^39^. In the present study, we confirmed the physical interaction between Cul7 and rEag1 channels in the brain, and showed that Cul7 and rEag1 exhibited significant co-localization at the synaptic region in neurons. Moreover, shRNA knock-down of endogenous Cul7 dramatically increases rEag1 protein levels in neurons, supporting a role of Cul7 in the regulation of rEag1 biosynthesis in the brain. Using the HEK293T heterologous expression system, we further demonstrated that Cul7, Fbw8, and rEag1 co-exist in the same protein complex, and that Cul7 over-expression leads to enhanced ubiquitination and reduced half-life of rEag1 channels. Overall, our observations are consistent with the idea that the Cul7 E3 ligase promotes rEag1 protein degradation.

One of the major findings of this report is that Cul7 degrades immature and mature rEag1 proteins via the proteasomal and the lysosomal pathways, respectively. Our biochemical analyses in HEK293T cells reveal that, in SDS-PAGE, rEag1 proteins manifest multiple electrophoretic mobility (protein bands a, b, and c) that reflect differential rEag1 glycosylation states. Previous studies have shown that human Eag1 comprises two N-linked glycosylation sites, N388 and N406, and that mutating either one of the two N-glycosylation sites prominently affects the stability and trafficking of Eag1 protein^40^. Accordingly, we observed two rEag1 protein bands with apparent molecular weights of about 110–120 kDa: the upper protein band (band a) corresponds to the mature, Golgi-processed, full-glycosylated rEag1 localized at the plasma membrane, whereas the lower protein band (band b) refers to the immature, core-glycosylated rEag1 that has yet to exit from the ER membrane during the early stage of biogenesis. Upon blocking proteasomal degradation with MG132, a third, lowest-molecular-weight rEag1 protein band (band c) emerges and appears to represent nascent, non-glycosylated rEag1 in the ER. As indicated by the Cul7 over-expression and knock-down experiments, Cul7 notably mediates degradation of rEag1 bands a and b, but perhaps not band c. Therefore, non-glycosylated rEag1 in the ER may be targeted for proteasomal degradation by an as yet unknown ubiquitin ligase. Nevertheless, given that ubiquitinated glycoproteins may be subject to deglycosylation by N-glycanase prior to proteasomal degradation^34^,^41^, we cannot rule out an alternative possibility that band c may also represent deglycosylated rEag1 rescued by the MG132 treatment.

We also presented morphological evidence showing that Cul7 over-expression enhances ER- and lysosome-localization of rEag1 in response to MG132 and chloroquine treatments, respectively. In addition, MG132 effectively reverses the effect of Cul7 on band b only, whereas the effect of Cul7 on band a is rescued by treatment with the lysosome blocker chloroquine. However, chloroquine notably rescues Cul7-mediated down-regulation of rEag1 band b as well. The significance of this observation requires further investigation. One possible interpretation is that a detectable portion of membrane rEag1 may be partially deglycosylated before being degraded by the endosome-lysosomal system. Alternatively, one may also argue that a fraction of immature, core-glycosylated rEag1 in the ER is targeted for lysosomal degradation.

We have previously demonstrated that rEag1 channels are significantly expressed at synapses, wherein rEag1 immunofluorescence signals display a distinct punctate localization pattern^10^,^12^. Similar punctate patterns were also reported by a different research group using single-particle-tracking techniques to characterize the presynaptic localization of rEag1 channels^13^. Moreover, by studying Eag1 knock-out mice, the same research group proposed that Eag1 appears to participate in the repolarization phase of action potentials and thereby regulate neurotransmitter release in the cerebellar circuit^42^. To the best of our knowledge, herein we presented the first immunofluorescence and subcellular fractionation evidence showing that, like Eag1, Cul7 is also abundantly expressed within synaptic regions. Given that ubiquitin-mediated proteolysis has been shown to control key proteins at both presynaptic terminals and postsynaptic compartments^26^,^43^, it remains to be determined whether the regulation of Eag1 protein level by Cul7 may play a significant role in modulating pre- and/or post-synaptic membrane excitability in the brain.

In addition to regulating membrane excitability, Eag1 has been suggested to play an important role during development, and several *KCNH1* gene mutations have been associated with the congenital neurodevelopmental anomalies TMBTS and ZLS^36^,^37^,^38^,^44^. TMBTS is characterized by intellectual disability, epilepsy, dysmorphic facial features, broad thumbs, and great toes with absent/hypoplastic nails. ZLS is characterized by facial dysmorphism including coarsening of the face and a large nose, gingival enlargement, intellectual disability, hypoplasia of terminal phalanges and nails, and hypertrichosis. Most of these disease-associated *KCNH1* mutations yield functional human Eag1 channels with gain-of-function phenotypes such as faster activation and slower deactivation kinetics. Some of the mutant Eag1 channels, however, display loss-of-function phenotypes such as diminished whole-cell currents. In this study, we demonstrated that a ZLS-associated, loss-of-function Eag1 mutant, G469R, exhibits enhanced protein degradation that can be partially rescued by suppressing endogenous Cul7 expression in HEK293T cells. Moreover, the G469R mutant is associated with a significant reduction in the proportion of mature, full-glycosylated form (band a) of rEag1, suggesting that a significant portion of rEag1-G469R is probably retained in the ER. Together, our data strongly suggest that the ZLS-associated arginine substitution at residue 469 may impose a grave interference of the ER protein folding process, leading to enhanced protein degradation as well as defective membrane trafficking of the mutant Eag1 channel.

The two mammalian Eag isoforms, Eag1 and Eag2, share about 70% identity in amino acid sequence, and both are widely expressed in various regions of the brain^4^,^7^. Like Eag1, the precise neurophysiological role of Eag2 channels remains elusive. Nevertheless, human Eag2 channel is known to facilitate cell motility of migrating medulloblastoma cells and may therefore contribute to tumor outgrowth and metastasis^45^,^46^. We found that Cul7 over-expression also significantly decreased the protein level of rat Eag2 proteins (Suppl. Fig. S7). It will be interesting to determine in the future whether Cul7 may play a role in the regulation of Eag2-mediated tumor migration.

Emerging evidence indicates that membrane-bound protein is susceptible to stringent conformation surveillance by both the ER and the peripheral quality controls^47^,^48^. Currently, it is unclear how misfolded or disease-associated mutant Eag1 proteins are recognized and processed by the two quality control systems. The results presented in this study indicate that Cul7 mediates both the ER and the peripheral protein degradations of rEag1 channels. Our findings may pave the way for future identification of additional components of the molecular machinery regulating Eag1 protein homeostasis in the ER and the plasma membrane, which in turn will provide a novel insight to the mechanisms underlying ER and peripheral protein quality controls of Eag1 channels.

## Methods

### cDNA constructs

rEag1 cDNA (Dr. Olaf Pongs, Saarland University) was subcloned into the pcDNA3.1 vector (Invitrogen). The G469R mutation was generated using the QuikChange site-directed mutagenesis kit (Stratagene), followed by DNA sequence verification. Other cDNA constructs include pcDNA3-Myc human cullin 3/4 A/4B/7 (Addgene 19893, 19951, 19922, 20695), pDsRed-Monomer-Membrane (Clontech), and pCR3.1-Myc human Fbw8 (Dr. Zhen-Qiang Pan, Mount Sinai School of Medicine).

### Cell culture and DNA transfection

Human embryonic kidney (HEK) 293 T cells were grown in DMEM (Invitrogen) supplemented with 10% fetal bovine serum (Hyclone) at 37 °C incubator with 5% CO_2_. Transient transfection was performed by the calcium phosphate method^49^. Cells were plated onto 6- or 12-well plates 24 hrs before transfection. The amount of rEag1 cDNA used in each well was about 200 (for expression) to 500 (for immunoprecipitation) ng/mL. The molar ratio for co-transfection was 2:1 (relative to rEag1 cDNA). Transfected cells were maintained at incubator for 24–48 hrs. Where indicated, cells were treated with ALLN (Sigma), brefeldin A (Sigma), chloroquine (Sigma), cycloheximide (Sigma), MG132 (Sigma), MLN4924 (Dr. Kuo-How Huang, National Taiwan University Hospital), or tunicamycin (Sigma).

### shRNA or siRNA knockdown

Small interference RNAs (siRNAs) (Invitrogen) include siCul7 #1 (CUL7HSS114814), siCul7 #2 (CUL7HSS114816), and negative control. Short hairpin RNAs (shRNAs) in the pLKO.1 puromycin-resistant vector (RNAi Core, Academia Sinica, Taiwan) include Cul7-shRNA (sh-Cul7#1: TRCN0000006480; sh-Cul7#2: TRCN0000006481) and GFP-shRNA (5′-CAACAGCCACAACGTCTATAT-3′). For lentiviral packaging in HEK293T cells, virus-containing supernatants were harvested and concentrated by ultracentrifugation to yield viral stocks, which were supplemented with 8 μg/ml of polybrene for lentiviral infection. Infected HEK293T cells were selected by 5 μg/ml of puromycin and subsequently transfected with rEag1 cDNA. For suppressing endogenous Cul7 in cultured neurons, DIV4 cortical neurons were infected with shRNA. After puromycin selection, infected DIV9 neurons were subject to immunoblotting.

### Immunoprecipitation and immunoblotting

Cultured cells or tissues were solubilized in ice-cold lysis buffer [(in mM) 100 NaCl, 4 KCl, 2.5 EDTA, 20 NaHCO_3_, 20 Tris-HCl, pH 7.5, 1 phenylmethylsulfonyl fluoride (PMSF)] with 1% Triton X-100, supplemented with protease inhibitor cocktail (Roche). Insolubilized materials were removed by centrifugation. Protein concentrations were determined by the BCA method (Thermo Scientific). For immunoprecipitation, solubilized lysates were pre-cleared with protein A/G Sepharose beads (GE Healthcare) and then incubated for 16 hrs at 4 °C with protein A/G Sepharose beads previously coated with desired antibodies. After washing with ice-cold lysis buffer, immune complexes were eluted from beads by boiling in the Laemmli sample buffer. For glycosidase treatment, solubilized cell lysates were treated with 1000 U N-glycosidase F (PNGase F) or endoglycosidase H (Endo H) (New England Biolabs) for 1 hr at 37 °C. Protein samples were separated on SDS-PAGE, immunoblotted with appropriate dilution of primary antibodies, and visualized with the ECL detection system (Advansta). Primary antibodies include anti-Myc (clone 9E10), anti-Cul7 (1:5,000; Sigma), anti-β-actin (1:10,000; Sigma), anti-cadherin (1:1,000; AbCam), anti-calnexin (1:1,000; Cell Signaling), anti-densin-180 (1:5000)^50^, anti-FK2 (1:1000; Enzo Life Sciences), GAPDH (1:1,000; GeneTex), anti-PSD95 (1:20,000; NeuroMab), anti-rEag1 and anti-rEag2 (1:5,000; Alomone Labs), and anti-synaptophysin (1:10,000)^10^. Immunoblot signals were quantified by using the ImageQuant software (GE Healthcare). Results shown are representative of at least 3 independent experiments.

### Cell surface biotinylation

Cells were rinsed with ice-cold PBS (in mM, 136 NaCl, 2.5 KCl, 1.5 KH_2_PO_4_, 6.5 Na_2_HPO_4_, pH 7.4) and incubated in 0.5 mg/mL sulfo-NHS-LC-biotin (Thermo Scientific) at 4 °C for 30 min with gentle shaking on an orbital shaker. After quenching the reaction, cells were solubilized with iced-cold lysis buffer and lysates were cleared at 10,000 × g for 5 min at 4 °C. Solubilized cell lysates were incubated for 16 hrs at 4 °C with streptavidin-agarose beads (Thermo Scientific). Biotin-streptavidin complexes were eluted from the beads by boiling for 5 min in the Laemmli sample buffer.

### Immunofluorescence

Cells grown on coverslips were rinsed in ice-cold D-PBS (PBS with 0.9 mM CaCl_2_, 0.5 mM MgCl_2_) and fixed for 20 min with 4% paraformaldehyde in PBS at 4 °C. After washing with cold PBS, fixed cells were permeabilized and blocked with a blocking buffer (5% normal goat serum in 20 mM phosphate buffer, pH 7.4, 0.1% (v/v) Triton X-100, and 0.45 M NaCl) for 60 min at 4 °C. Cells were then immunolabeled with the following primary antibodies at 4 °C for 16 hrs: rabbit anti-rEag1 antibody (Alomone; 1:1000), mouse anti-Myc antibody (clone 9E10, 1:100), mouse anti-calnexin antibody (1:200; Cell Signaling), or mouse anti-lamp1 antibody (1:500; Abcam). Alexa Fluor 488-conjugated rabbit IgG and Alexa Fluor 568-conjugated mouse IgG (1:200; Molecular Probes) were used as secondary antibodies. Nuclei were stained with DAPI. After final wash, coverslips were mounted in a mounting medium (4% n-propylgallate, 90% glycerol, 0.1 M carbonate buffer, pH 9.2), and observed using a TCS SP2 laser-scanning microscope (Leica) equipped with a 63x oil immersion objective.

### Animals and neuronal cultures

All animal procedures were in conformity with the animal protocol approved by the Institutional Animal Care and Use Committee (IACUC) of National Yang-Ming University. Dissociated neuronal cultures were prepared from embryonic embryos (E18 or E19) of Sprague-Dawley rats^51^. Dissociated cortical and hippocampal neurons were plated at 1000 and 200 cells/mm^2^. Neurons were maintained in the Neurobasal (Invitrogen) supplemented with 2% B27 (Invitrogen) and 2 mM glutamine in a humidified 5% CO_2_ incubator at 37 °C.

### Preparation of brain homogenates, synaptosomes and PSD fractions

For synaptosomes and PSD preparation, rat forebrains were homogenized in buffer H1 [(in mM) 320 sucrose, 1 NaHCO_3_, 0.5 CaCl_2_, 0.1 PMSF] containing the complete protease inhibitor cocktail (Roche). Tissue homogenates were centrifuged at 1,400 × *g* for 10 min to recover the supernatant S1 and the pellet P1. The S1 fraction was subjected to centrifugation at 13,800 × *g* for 10 min to obtain the P2 pellet. Pellets were resuspended in buffer H2 [(in mM) 0.32 M sucrose and 1 mM NaHCO_3_)] and layered onto the top of discontinuous sucrose density gradient with 0.85, 1.0, and 1.2 M sucrose layers. Gradients were centrifuged at 65,000 × *g* for 2 hrs in a Beckman SW-28 rotor. Synaptosomal (SPM) fractions were recovered from the 1.0–1.2 M sucrose interface and extracted in ice-cold 0.5% Triton X-100/50 mM Tris-HCl (pH 7.9) for 15 min and centrifuged at 32,000 × *g* for 45 min to obtain the PSD I pellet. PSD I pellets were resuspended and further extracted a second time with 0.5% Triton X-100/50 mM Tris-HCl (pH 7.9), followed by centrifugation at 200,000 × *g* for 45 min to obtain the PSD II pellet.

### Differential centrifugation and sucrose gradient fractionation

Cells were washed with iced-cold PBS and detached from petri dishes with PBS and 2 mM EDTA. Cells were pelleted for 5 min at 250 × *g*. Cell pellets were resuspended in hypotonic buffer [(in mM) 20 Tris-HCl, pH 7.9, 2 dithiothreitol, 10 EDTA, and 1 PMSF supplemented with protease inhibitor cocktail (Roche)], homogenized by 20 strokes in a Dounce homogenizer and centrifuged at 250 × *g* at 4 °C for 10 min to removed undisrupted cells. Pellets were discarded and homogenates were supplemented with 2.0 M sucrose to achieve a 0.2 M final sucrose concentration. After 90 min centrifugation at 100,000 × g, supernatant and pellet fractions contained cytosolic and membrane proteins, respectively. Pellets were rinsed with hypotonic buffer and resuspended to the same volume as supernatants in iced-cold lysis buffer [(in mM) 100 NaCl, 4 KCl, 2.5 EDTA, 20 NaHCO_3_, 20 Tris-HCl, pH 7.5, 1 PMSF] with 1% Triton X-100.

For sucrose gradient fractionation, we employed a previously published protocol with some modifications^52^. Cells were lysed and ultracentrifuged to separate the cytosol and the membrane fractions as describe above. Membrane pellet fractions were suspended in hypotonic buffer supplemented with 2.0 M sucrose to achieve a 0.2 M final sucrose concentration. Membrane lysates were loaded on the top of a discontinuous sucrose gradient [2.0 M (0.4 ml), 1.5 M (0.75 ml), 1.35 M (0.75 ml), 1.2 M (0.75 ml), 0.9 M (0.5 ml), and 0.5 M (0.5 ml) in hypotonic buffer], and subject to a 32,000 × *g* centrifugation for 16 hrs in a Beckman SW55 rotor. Fractions were collected from the top of the gradient, including one 1.0-ml and seven 0.5-ml fractions.

### Electrophysiology

Whole-cell patch clamp recordings of rEag1 K^+^ currents in HEK293T cells (24 hrs post-transfection) were performed at room temperature (20–22 °C). Electrode solution contained (in mM) 140 KCl, 1 MgCl_2_, 10 EGTA, 10 HEPES, pH 7.2. Bath solution comprised (in mM) 140 NaCl, 5 KCl, 1 CaCl_2_, and 10 HEPES, pH 7.2. Data were acquired with an Axopatch 200 A amplifier (Molecular Devices) and digitized with the Digidata 1420 A system and the pCLAMP 10.2 software (Molecular Devices). Cells with large currents in which voltage clamp errors might appear were excluded from data analyses. Passive membrane properties were compensated by using the −P/4 leak subtraction method. Data were sampled at 10 kHz and filtered at 1 kHz.

### Statistical analysis

Data are presented as mean ± SEM. The significance of the difference between two means was tested by Student’s *t*-test, whereas means from multiple groups were compared by one-way ANOVA. All statistical analyses and curve fitting were performed with the Origin 7.0 software (Microcal).

## Additional Information

**How to cite this article**: Hsu, P.-H. *et al*. Cullin 7 mediates proteasomal and lysosomal degradations of rat Eag1 potassium channels. *Sci. Rep.* **7**, 40825; doi: 10.1038/srep40825 (2017).

**Publisher's note:** Springer Nature remains neutral with regard to jurisdictional claims in published maps and institutional affiliations.

## Supplementary Material

Supplementary Information
