# Supplementary material for: Cullin 7 mediates proteasomal and lysosomal degradations of rat Eag1 potassium channels
Source: Sci Rep. 2017 Jan 18;7:40825. doi: 10.1038/srep40825 (PMC5241692; doi:10.1038/srep40825)

**Supplementary Information**

**Cullin 7 mediates proteasomal and lysosomal degradations of rat Eag1 potassium channels**

Po-Hao Hsu, Yu-Ting Ma, Ya-Ching Fang, Jing-Jia Huang, Yu-Ling Gan, Pei-Tzu Chang, Guey-Mei Jow, Chih-Yung Tang, and Chung-Jiuan Jeng

**SUPPLEMENTARY METHODS**

***Yeast two-hybrid screening***

The cDNA sequence corresponding to the carboxyl-terminal region of rEag1 (amino acids 493-962, also known as the C0 fragment) was amplified by PCR and fused in-frame to the coding sequence for the DNA-binding protein LexA in the yeast expression plasmid pGilda (OriGene). The rEag1 C0 fragment was used as the bait to screen a rat brain cDNA library subcloned in the plasmid pJG4-5 (OriGene) by employing the DupLEX-A yeast two-hybrid system (OriGene). Briefly, the yeast strain EGY48, which contains the reporter gene LEU2 downstream from the LexA-operator, was sequentially transformed (using the lithium acetate method) with *i*) the bait-containing plasmid pGilda, *ii*) the reporter plasmid pSH18-34 (containing the LexA operator-lacZ fusion gene), and *iii*) the activation domain-fused cDNA library in the plasmid pJG4-5. After incubating at 30˚C for 2-7 days, transformed yeast colonies growing on leucine dropout plates were scored positive for interacting proteins. Positive colonies were further selected by the β-galactosidase assay. Plasmid DNA was extracted from yeast colonies and used to transform the *E. coli* strain DH5α. Candidate cDNA clones were screened by PCR with pJG4-5-specific primers, followed by online (BLAST) and in-house sequence analyses.

To ensure that the rEag1 C0 fragment-LexA fusion protein was effectively expressed in EGY48, transformed EGY48 yeast colonies were in addition cultured at 30°C in 4 ml of YPD rich media, harvested just before colony density reached the OD_600_ value 0.6, lysed in 20 μl of B-PER reagent (Pierce), and examined by immunoblotting analyses with the anti-LexA and the anti-rEag1 antibodies (Suppl. Fig. S1A).

***Glutathione S-transferase (GST) pull-down assays***

GST fusion proteins were produced and purified by following the manufacturer’s instruction (Stratagene). In brief, cDNA fragments encoding six different carboxyl-terminal regions of rEag1 (Suppl. Fig. S1B) were subcloned into the pGEX vector (GE Healthcare) and expressed in the *E. coli* strain BL21*.* Bacterial cultures were grown at 30°C, induced with 0.1 mM isopropyl-β-D-thiogalactopyranoside, and then harvested by centrifugation at 8,000x*g* for 10 min at 4ºC. Bacterial cell pellets were resuspended in the B-PER reagent containing 1 mM phenylmethylsulfonyl fluoride (PMSF) and protease inhibitor cocktail (Roche Applied Science). The bacterial lysates were clarified by centrifugation at 15,000x*g* for 15 min, and glutathione-agarose beads (Sigma) were used to bind the GST fusion proteins from the supernatant. GST protein-coated beads (4–8 μg) were incubated with pre-cleared HEK293T cell lysates at 4ºC overnight. The bead-protein complexes were then washed with buffer A [(in mM) 100 NaCl, 4 KCl, 2.5 EDTA, 20 NaHCO_3_, 20 Tris-HCl, pH 7.5, plus 1 PMSF, 1 Na_3_VO_4_, 1 NaF, 1 β-glycerophosphate] (with and without 1% Triton X-100). The proteins were eluted by boiling for 5 min in the Laemmli sample buffer, followed by immunoblotting analyses with the anti-Myc and the anti-GST antibodies (Suppl. Fig. S1C).

***Image analyses of immunofluorescence data***

Image analyses were performed with the ImageJ software (National Institute of Health).

To estimate the number of immunofluorescence clusters per fixed length of neurites in cultured hippocampal neurons (see Fig. 2E), built-in “set scale” and “freehand tool” functions of ImageJ were employed to trace multiple 100-μm neurite segments, followed by counting the number of Cul7/densin/rEag1 puncta within each 100-μm neurite segment (Suppl. Fig. S2A). Co-localization of Cul7 (see green punctate pixels in Fig. 2E) with densin/rEag1 (see red punctate pixels in Fig. 2E) puncta was expressed as the fraction of puncta that are identified as both green and red punctate pixels within each 100-μm neurite segment (Suppl. Fig. S2A).

To assess the effect of Cul7 co-expression on the subcellular localization of rEag1 in HEK293T cells (see Fig. 7), we determined the co-localization of rEag1 with DsRed-Mem (see Fig. 7B), calnexin (see Fig. 7F), or Lamp1 (see Fig. 7I) by using the built-in Coloc 2 module of ImageJ, wherein the autothreshold function was applied to evaluate the degree of pixel co-localization within a region of interest by calculating Pearson’s correlation coefficient (ranging from -1 to 1). A Pearson’s correlation coefficient value of 0.5 or higher is conventionally considered as a significant degree of co-localization.

***Supplementary Figure S1. Interaction of Cul7 with rEag1 carboxyl-terminal region.*** (related to Figure 1)

(A) Representative immunoblot showing the expression of LexA-rEag1-C0 fusion protein in EGY48 yeasts transformed with the bait-containing pGilda-rEag1-C0 plasmid, but not in those transformed with the pGilda vector *per se*. Protein samples (10 μl) were examined by immunoblotting (WB) with the anti-LexA and anti-rEag1 antibodies. (B) Schematic representation of the domain structure of the six GST-rEag1 fusion proteins employed for pull-down experiments. (C) The rEag1 CNBHD region serves as the principal Cul7-interacting domain. Cell extracts from HEK293T cells transfected with Myc-Cul7 were subject to GST pull-down assay with GST or GST fusion proteins comprising six different rEag1 carboxyl-terminal regions (GST-C0, GST-C1, GST-C2, GST-C3, GST-C1-A and GST-C1-B). Pull-down products were detected by immunoblotting with anti-Myc antibody. GST and GST-rEag1 fusion proteins were detected by immunoblotting with the anti-GST antibody. Input represents 5% of total cell lysate volume. Arrowheads denote the location of GST or GST-rEag1 fusion protein bands.


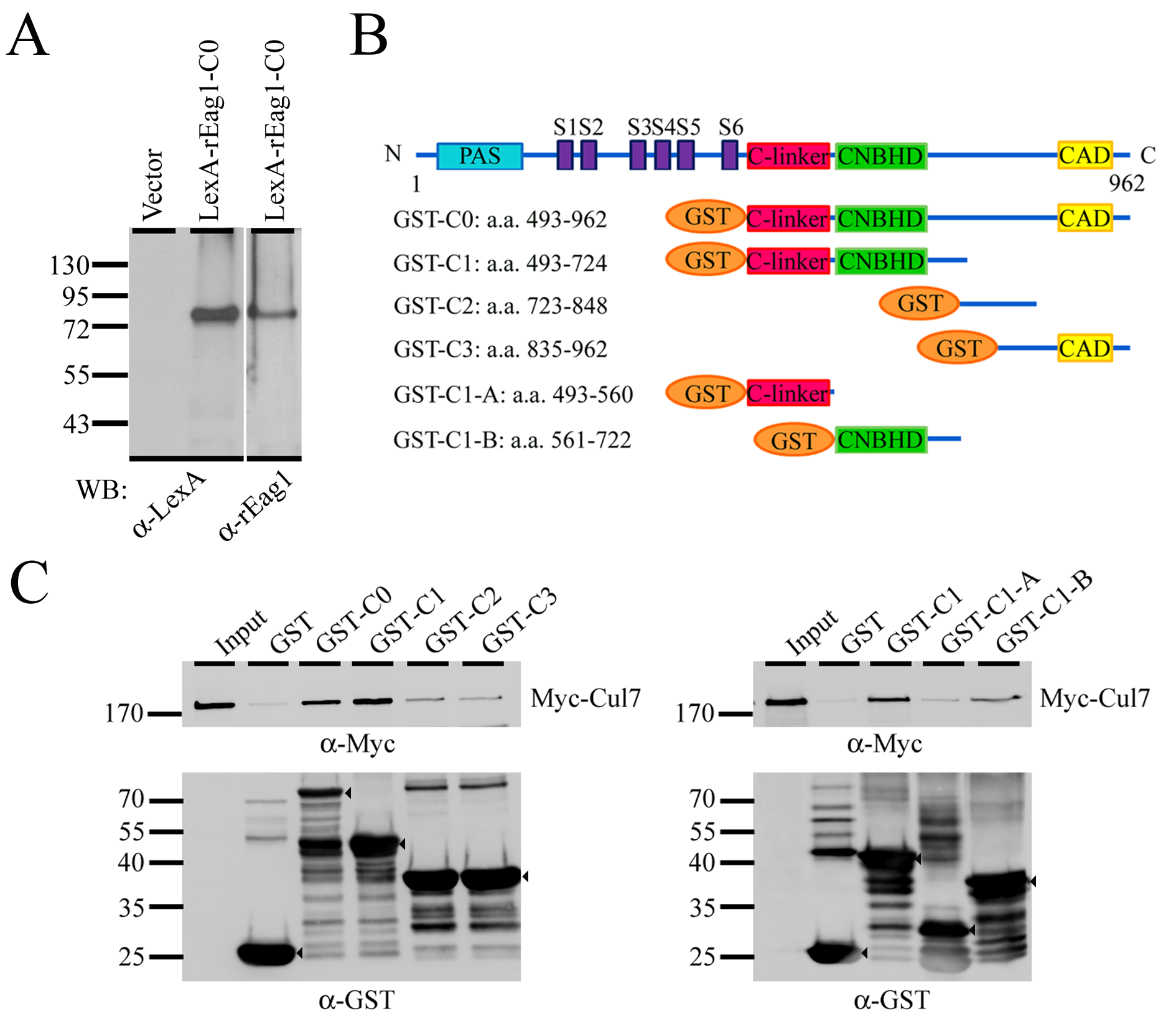


***Supplementary Figure S2. Quantification of immunofluorescence images.*** (related to Figure 2E and Figure 7)

(A) Quantification of the number of puncta per 100-μm neurite (puncta/100 μm) and the fraction of puncta co-localization in cultured hippocampal neurons (see Fig. 2E). (*Left panels*) Analyses of densin and Cul7 puncta in 27 different neurites associated with 5 different neurons. (*Right panels*) Analyses of rEag1 and Cul7 puncta in 25 different neurites associated with 7 different neurons. Co-localization is expressed as the fraction of densin/rEag1 puncta co-localized with Cul7 puncta, as well as the fraction of Cul7 puncta co-localized with densin/rEag1 puncta. (B) Quantification of the co-localization of rEag1 with DsRed-Mem (see Fig. 7B), calnexin (in the presence of MG132; see Fig. 7F), or lamp1 (in the presence of chloroquine; see Fig. 7I) in HEK293T cells in the absence (vector control) or presence of Cul7. The data represent the average of Pearson’s correlation coefficient values calculated from 6-11 different cells. Asterisks indicate significant difference (*t*-test, p<0.05) from the corresponding vector control.


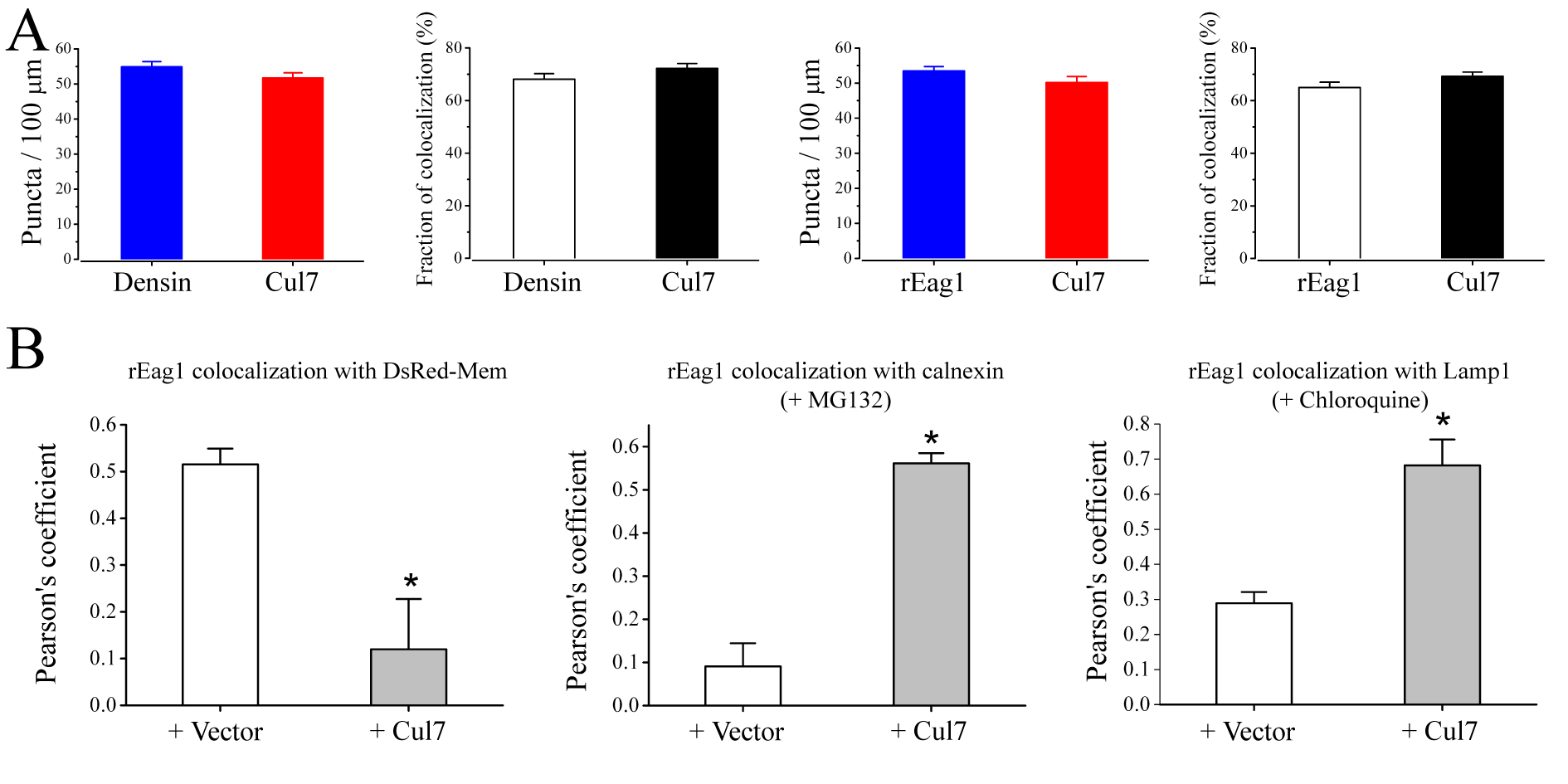


***Supplementary Figure S3. Cul7 promotes rEag1 protein degradation in HEK293T and B35 cells.*** (related to Figure 3)

(A) 12-hour treatment with 10 μM MLN4924 (*MLN*) up-regulates rEag1 protein expression and reverses the effect of Cul7 co-expression on rEag1. (B) shRNA knock-down of endogenous Cul7 expression enhances rEag1 protein level in B35 rat neuroblastoma cells. rEag1 or Cul7 signals were standardized as the ratio to the cognate GAPDH levels, followed by normalization to the corresponding DMSO/shGFP control (*, p<0.05; **, p<0.01; n=5). The gels were run under the same experimental conditions.


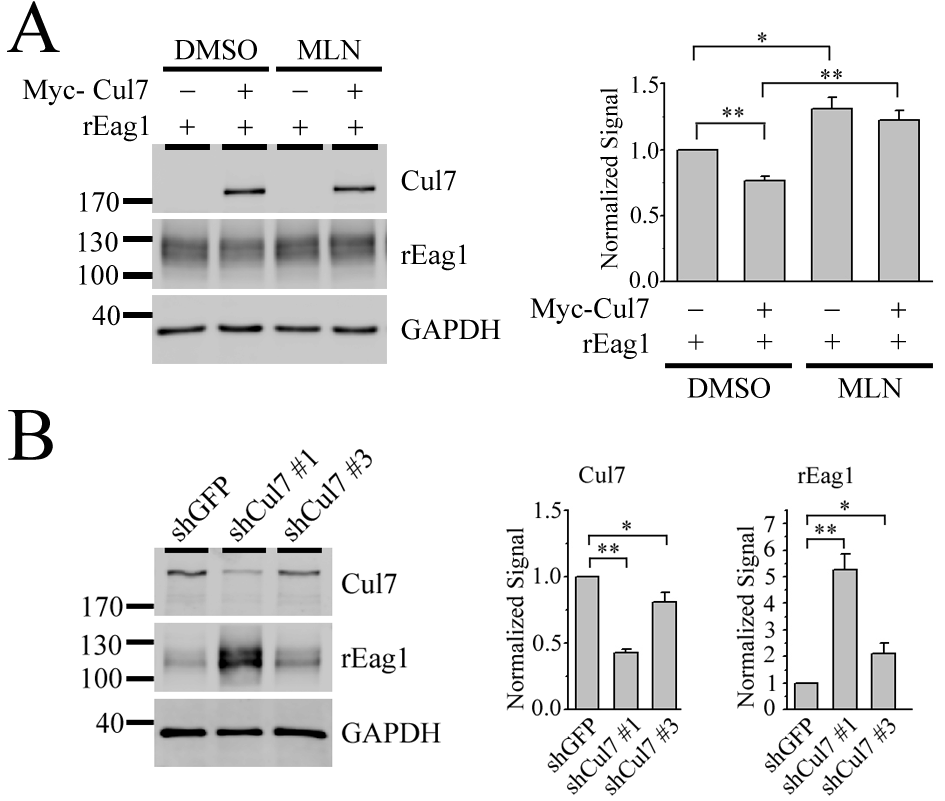


***Supplementary Figure S4. Cul7 reduces rEag1 current expression without affecting the channel’s steady-state voltage dependence property.*** (related to Figure 3G)

(*Left panel*) Normalized current density–voltage (Norm. I-V) relationship of rEag1 channels in the absence or presence of Cul7. The data points at +60 mV correspond to the right panel in Figure 3G. (*Right panel*) Steady-state activation (*P_o_*–V) curves of rEag1 channels in the absence or presence of Cul7. Isochronal tail currents at -90 mV in response to various test pulse potentials were fit with a Boltzmann function: Po(V) = 1/{1+exp[(V_0.5_-V)/*k*]}, where V_0.5_ is the half-maximal voltage for activation, and *k* the slope factor [n = 19 (vector), 14 (Cul7)].


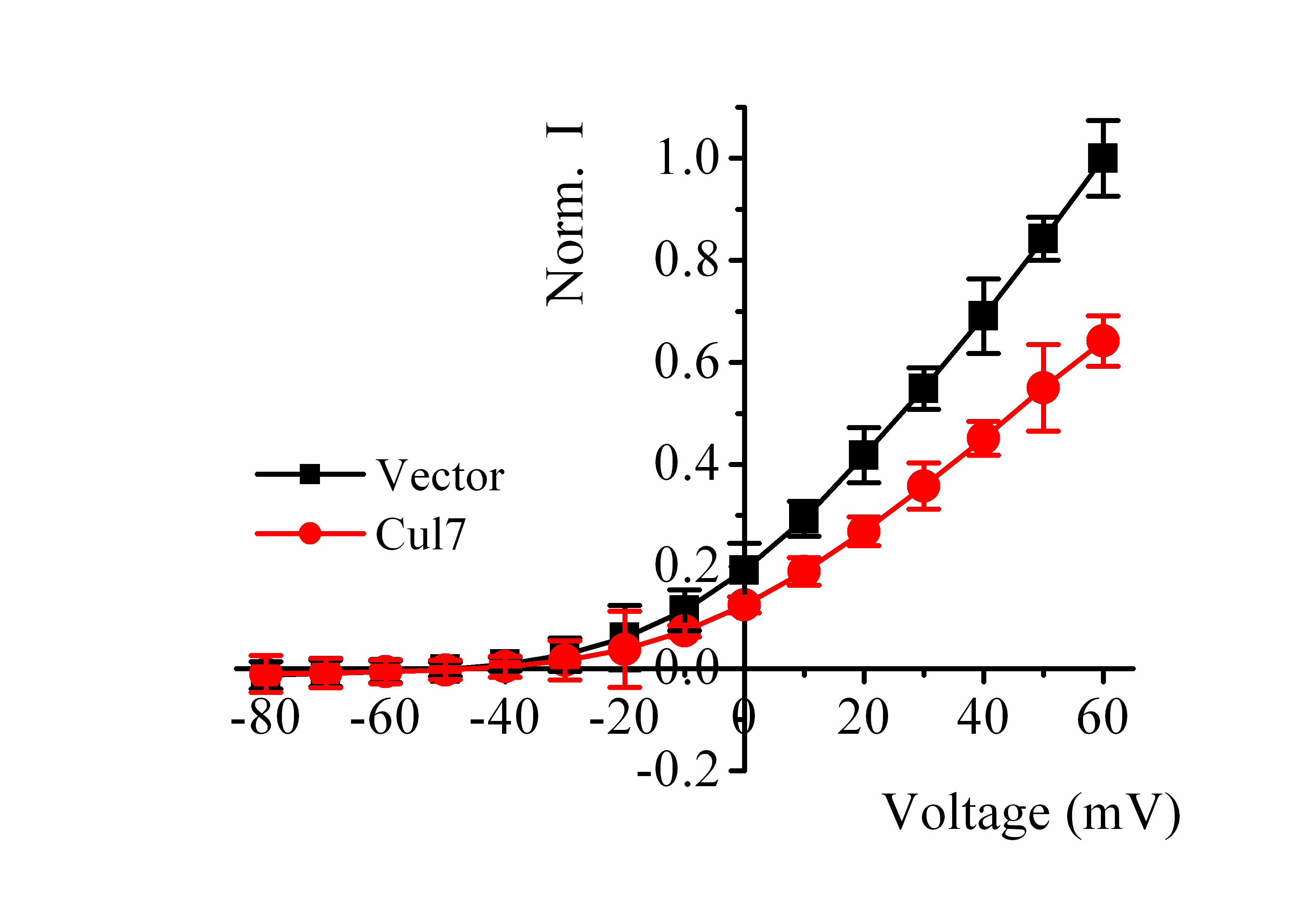

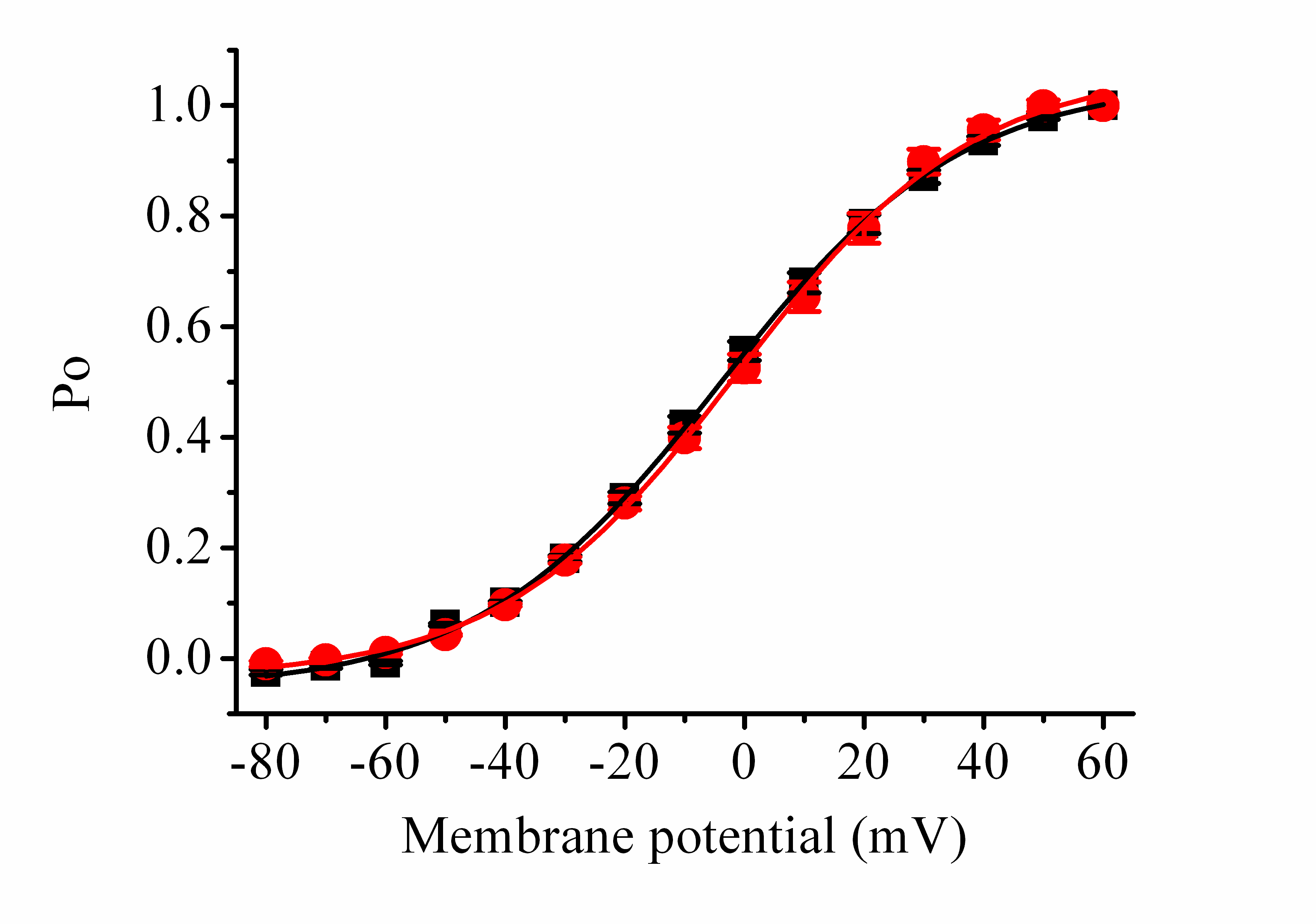


***Supplementary Figure S5. Cul7 promotes proteasomal degradation of rEag1.*** (related to Figure 5)

(A) Schematic representation of how we determined the region of interest (pink squares) for the quantification of rEag1 protein bands a, b, and c. Shown here is an enlarged version of the immunoblot presented in the left panel of Figure 5D, which exemplifies the effect of MG132 treatment on Cul7 regulation of various rEag1 protein bands. (B) Cul7 co-expression does not appreciably affect rEag1 protein ubiquitination in the absence of MG132 and chloroquine (MG132 −). Ubiquitinated rEag1 was visualized as protein smears with high molecular weights. The numbers denote quantification of relative ubiquitinated rEag1 levels with respect to the corresponding vector control. The gels were run under the same experimental conditions.


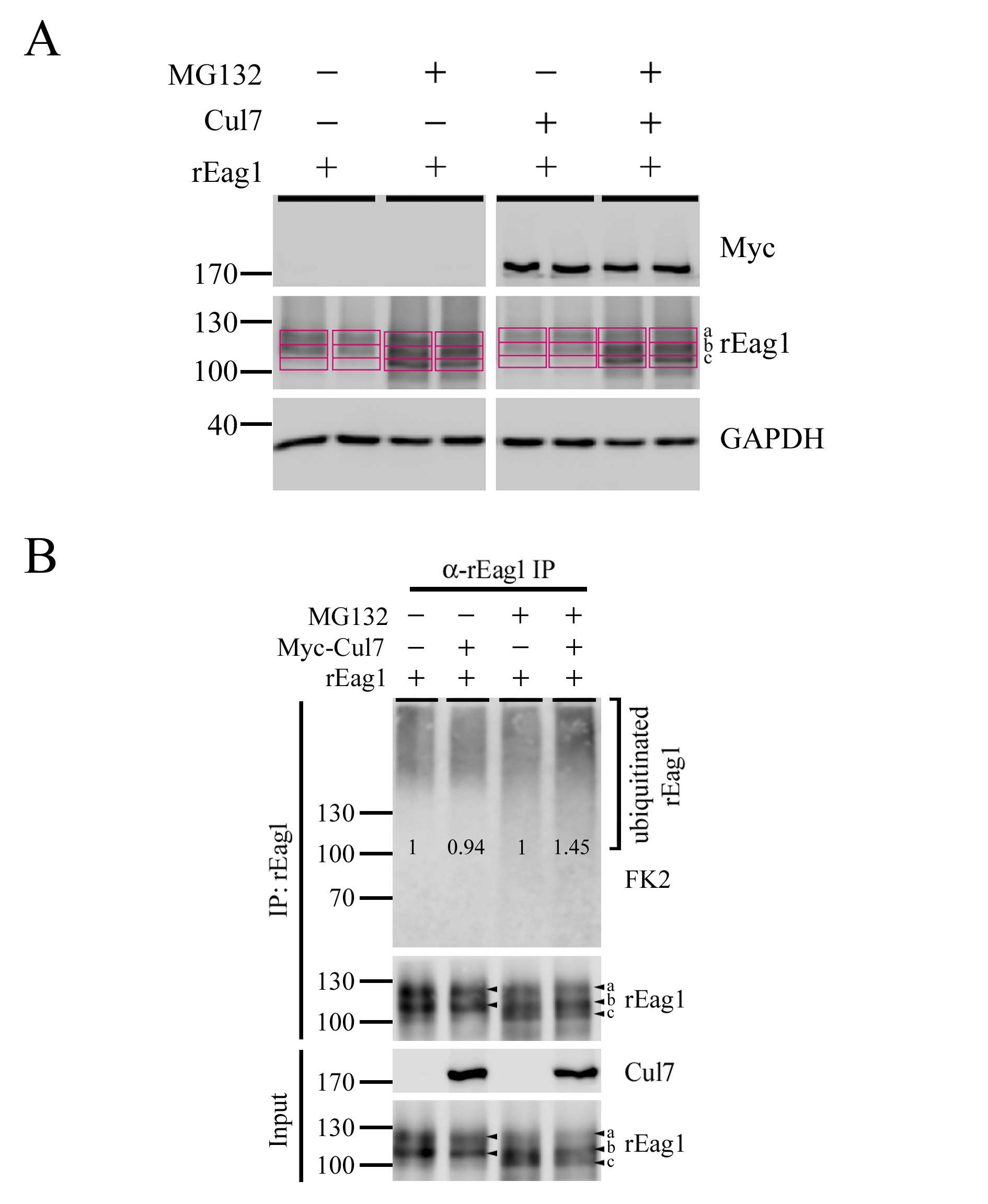


***Supplementary Figure S6. Cul7 promotes protein degradation of surface rEag1.*** (related to Figure 6)

The turn-over time course of cell surface rEag1 protein in HEK293T cells was determined with the Golgi trafficking inhibitor brefeldin A (BFA). (A) Representative immunoblots showing the effect brefeldin A treatment in the absence or presence of Cul7. Prolonging brefeldin A treatment duration leads to reduction and enhancement of rEag1 protein bands a and b signals, respectively. (B) Quantification of brefeldin A-induced change in rEag1 protein bands a (*left*) and b (*right*) signals in the absence or presence of Cul7. Protein densities were standardized as the ratio of rEag1 signals to the cognate GAPDH signals, followed by normalization to those of the corresponding no-treatment control at 0 hr. Data points represent the average of 4 independent experiments. (C) Determination of the protein half-life of rEag1 protein band a in the absence or presence of Cul7. (*Left*) Linear-regression analyses (solid lines) of the semi-logarithmic plot of rEag1 protein band a degradation time course as shown in the left panel in (B). *(Right)* Statistical analyses of rEag1 protein half-life in the absence or presence of Cul7 (*, p<0.05; n=4). The protein degradation time course determined from each experiment was individually plotted on a semi-logarithmic scale for linear-regression analyses.


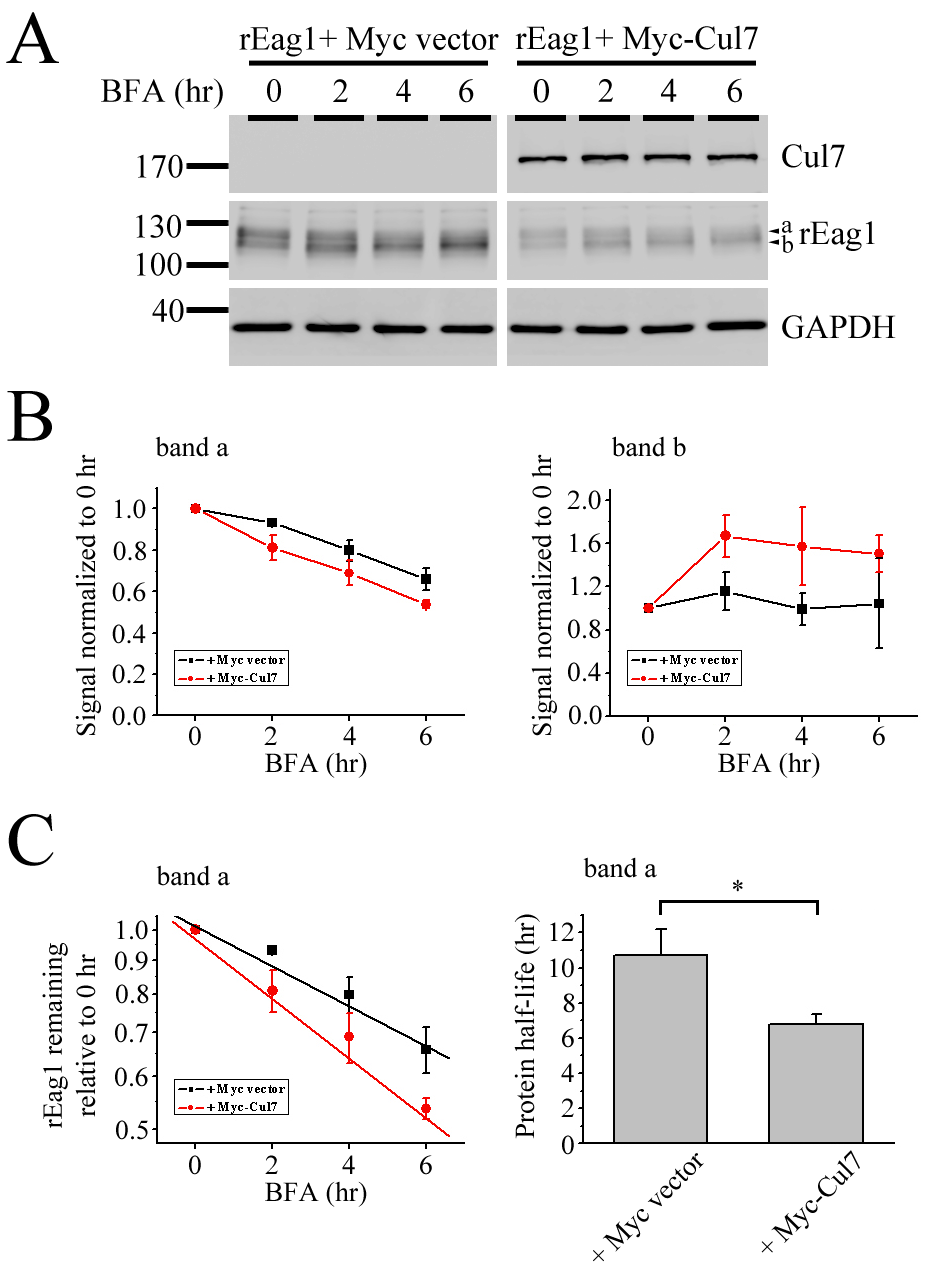


***Supplementary Figure S7. Cul7 mediates protein degradation of rEag2.*** (related to Discussion)

rEag1/rEag2 was transiently co-transfected with the Myc vector or Myc-Cul7 into HEK293T cells. 48 hours after transfection, cells were lysed and processed for immunoblotting. Cul7 over-expression significantly decreases the expression level of rEag1 (A) and rEag2 (B) proteins (*, p<0.05; **, p<0.01; n = 3). The gels were run under the same experimental conditions.


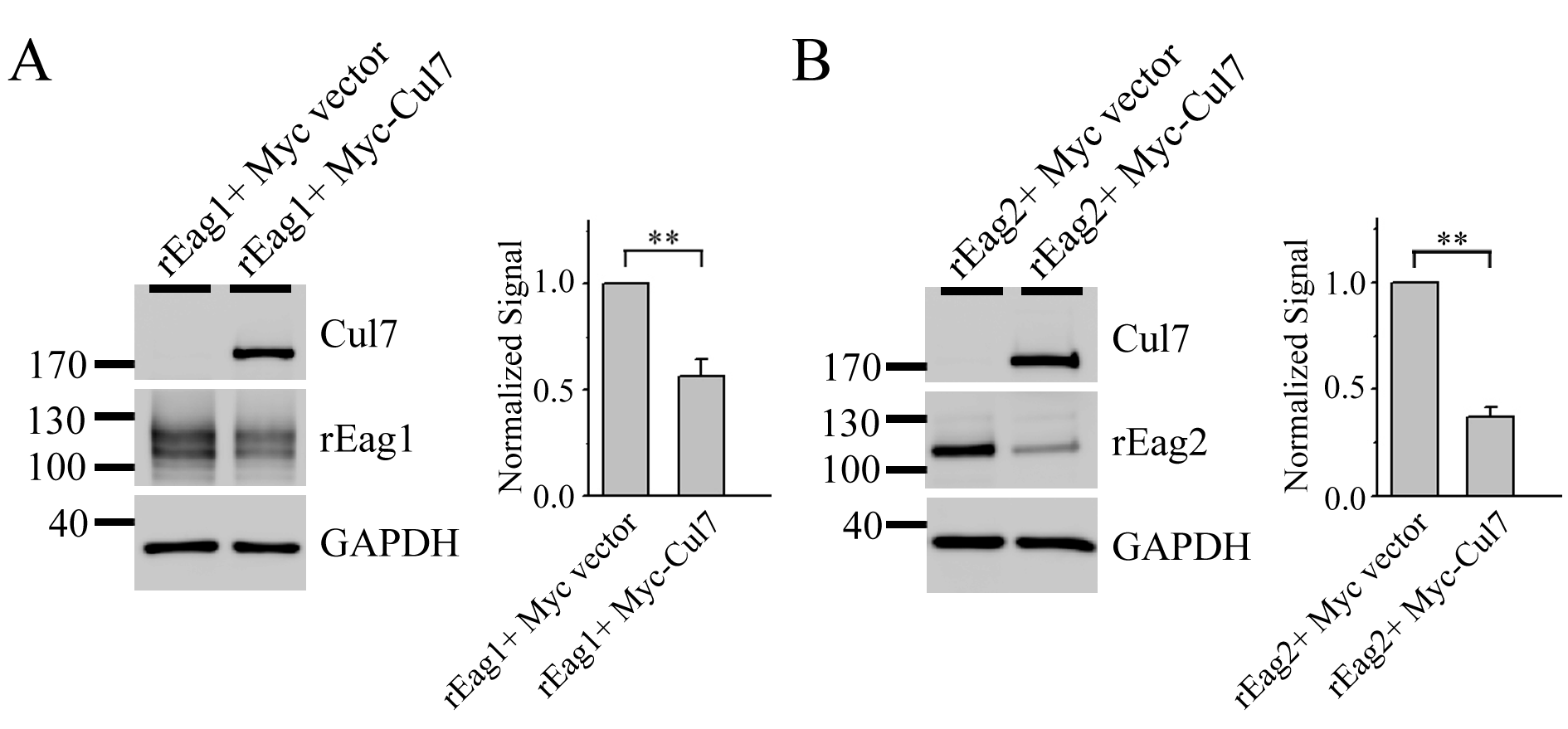


***Supplementary Figure S8. Uncropped images of the immunoblots presented in the main figures.***

(related to Figure 1)


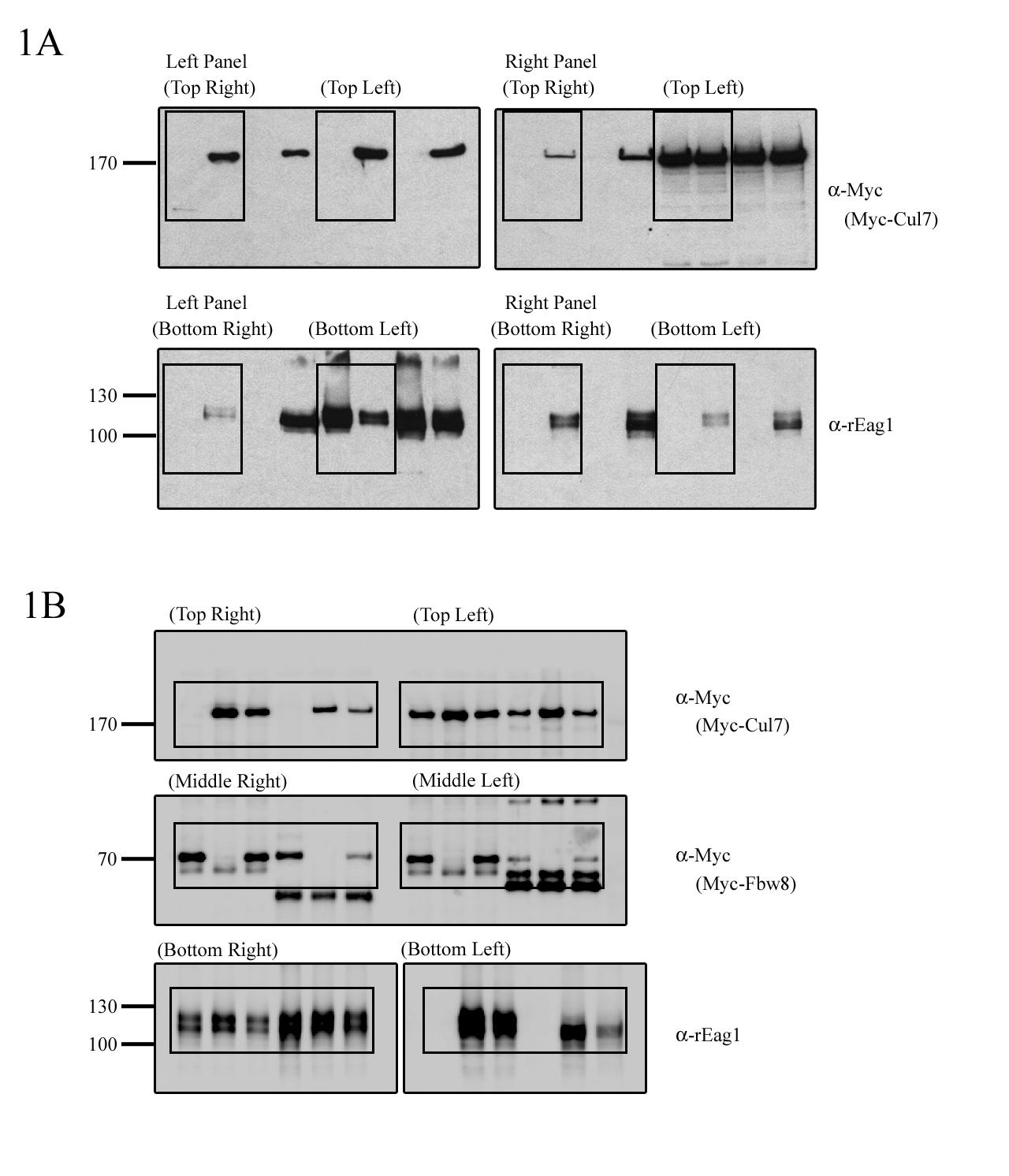


(related to Figure 2)


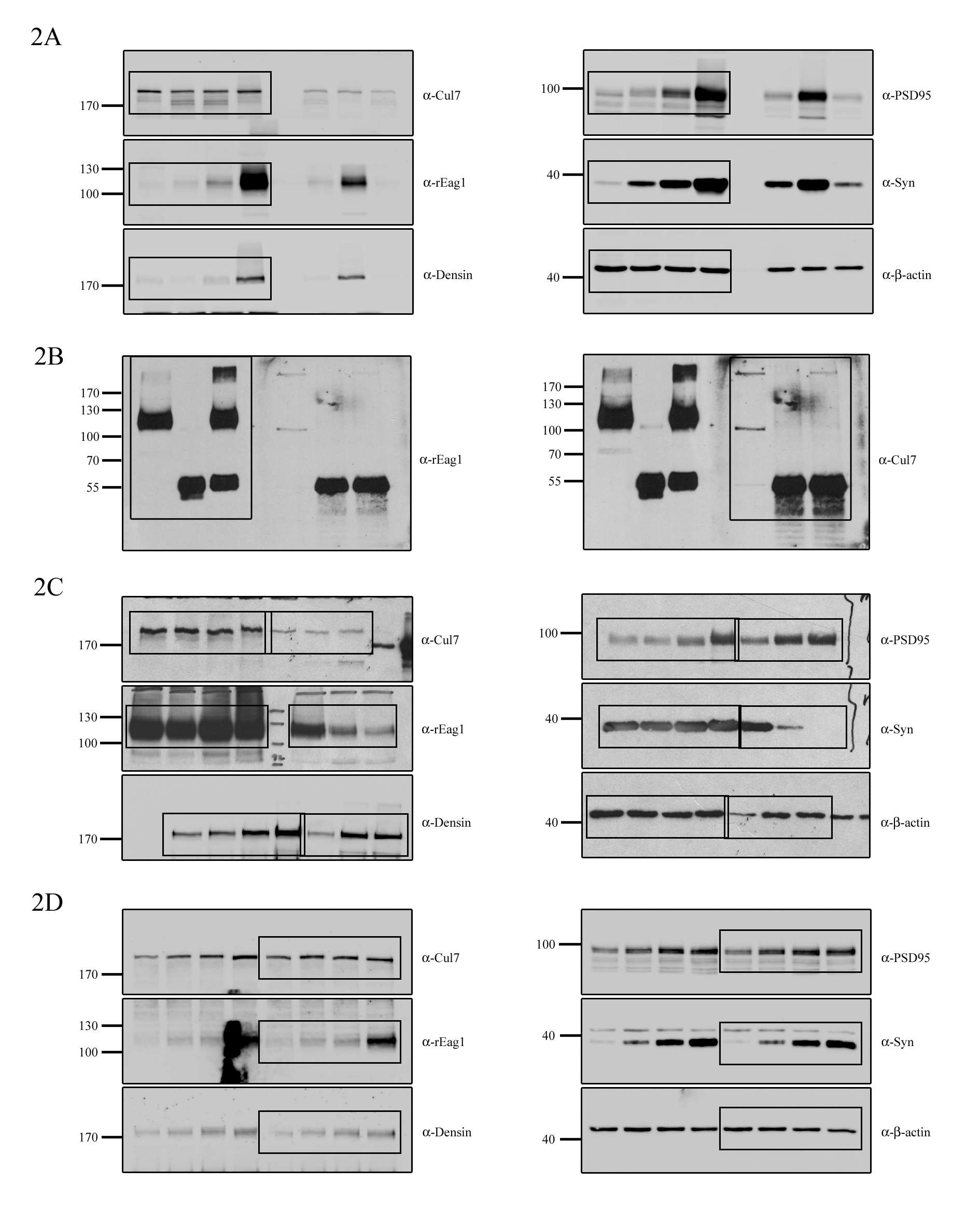


(related to Figure 3)


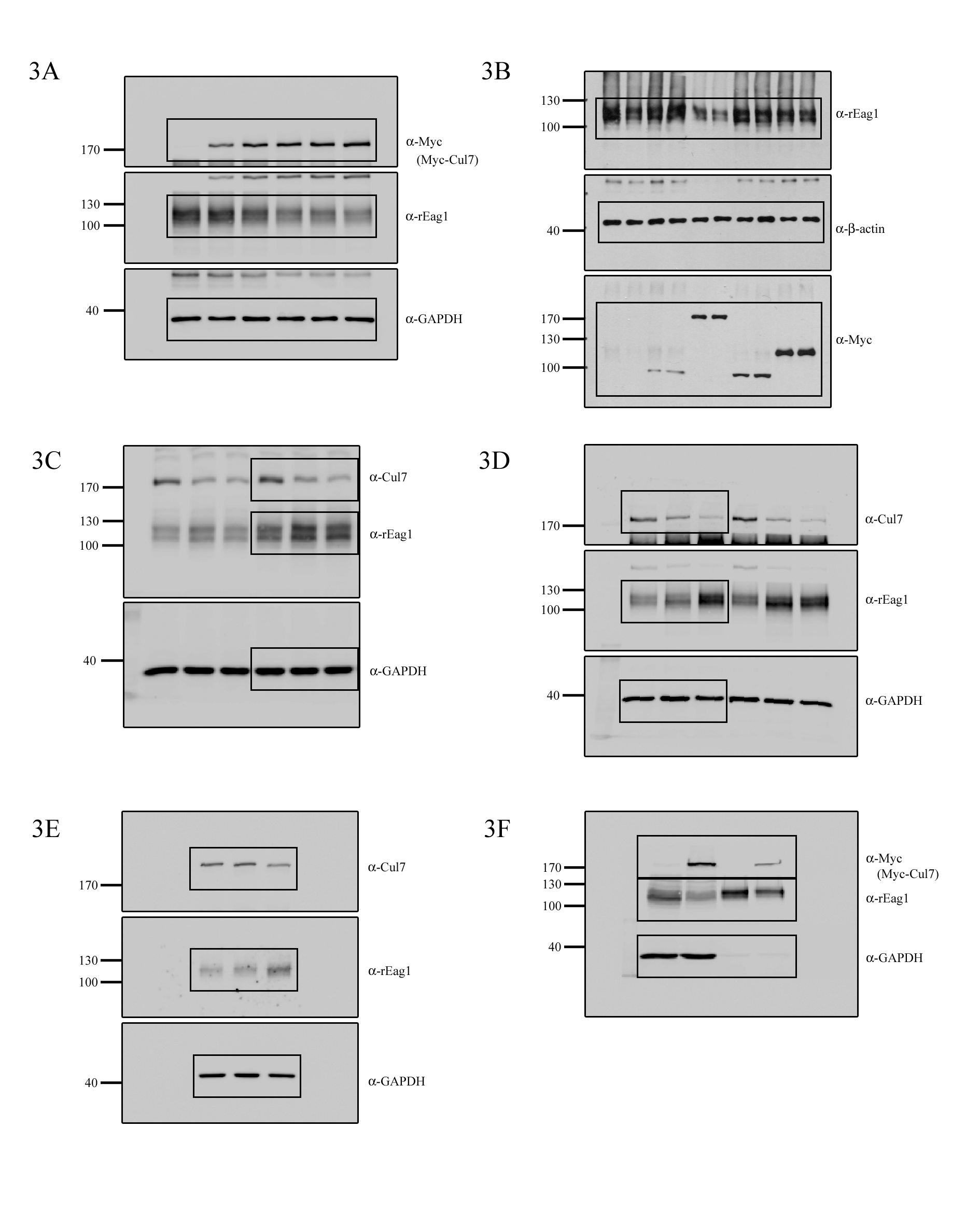


(related to Figure 4)


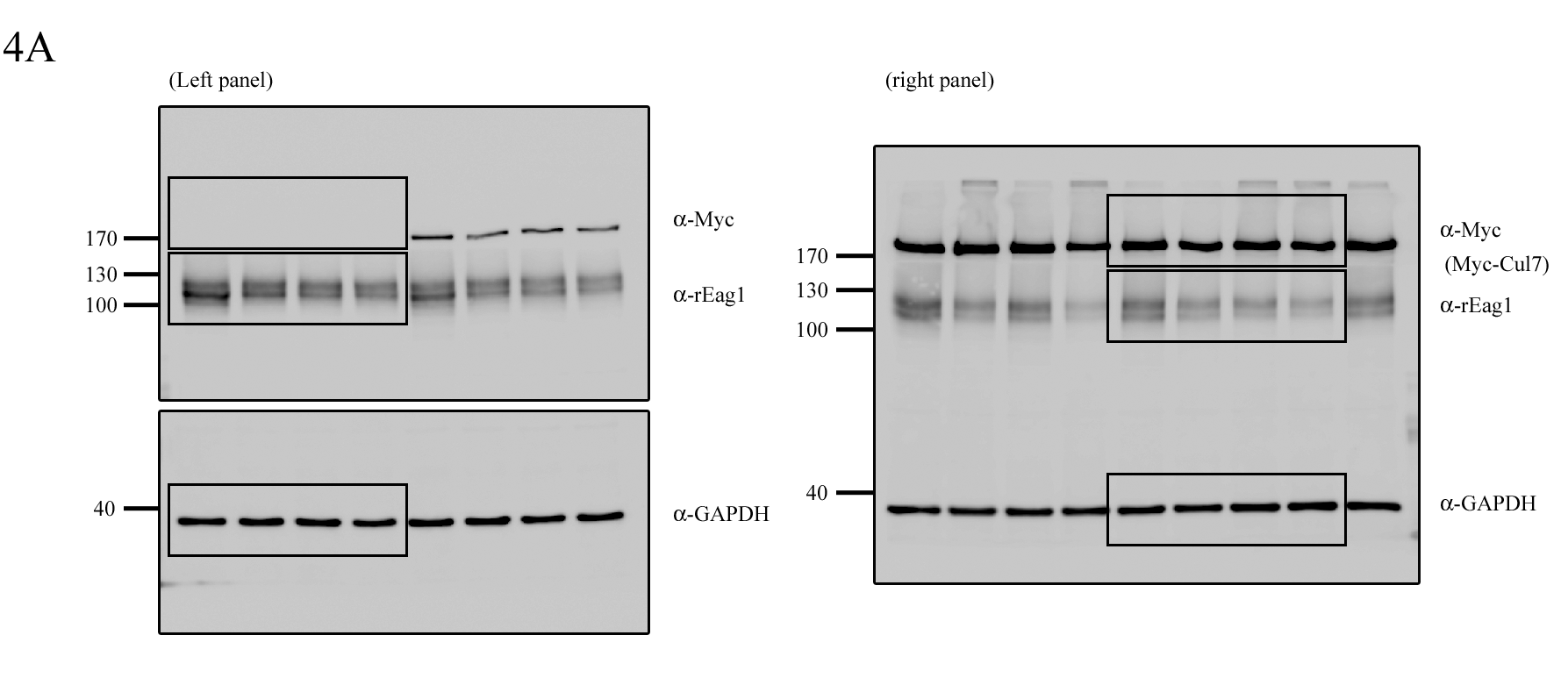


(related to Figure 5)


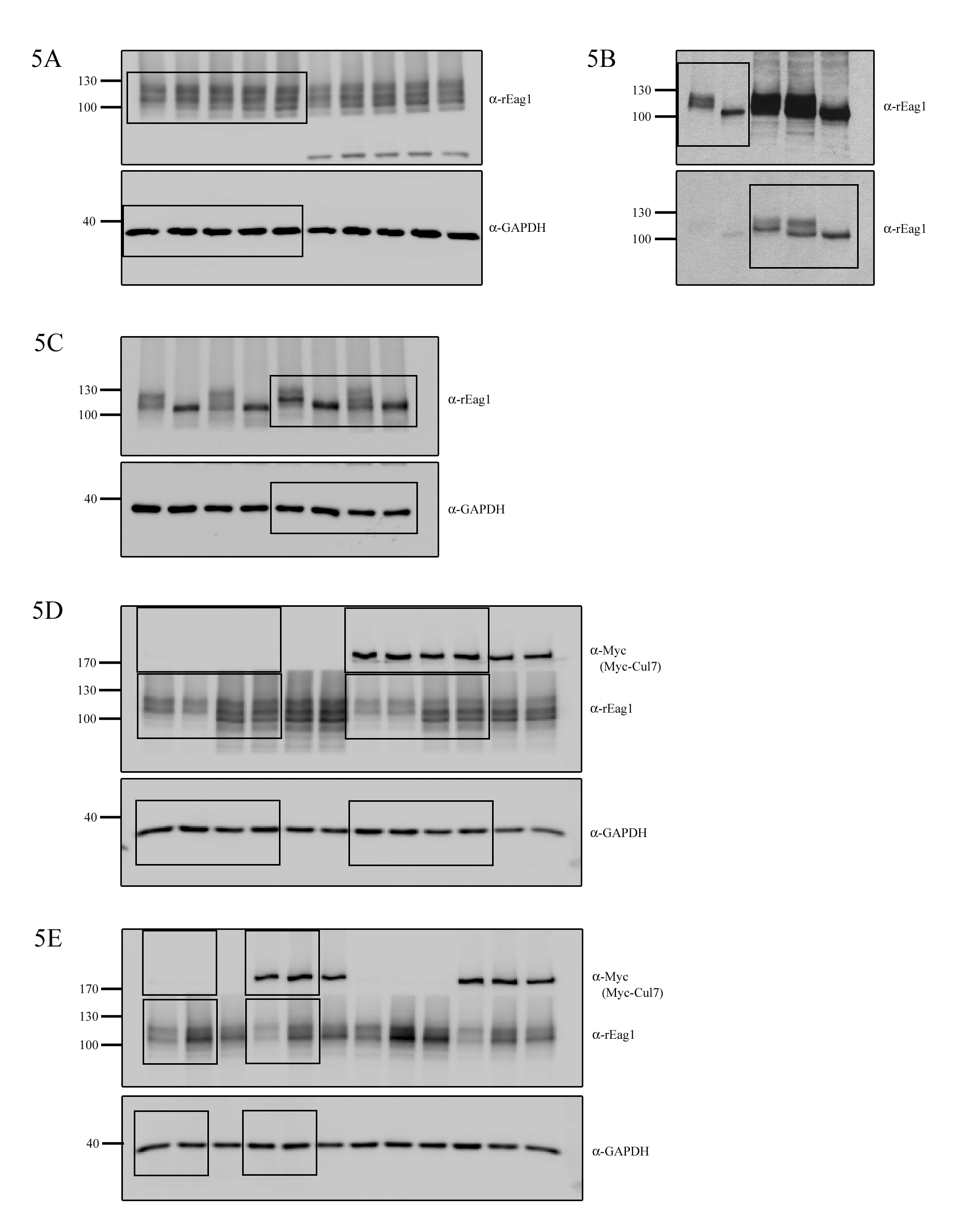


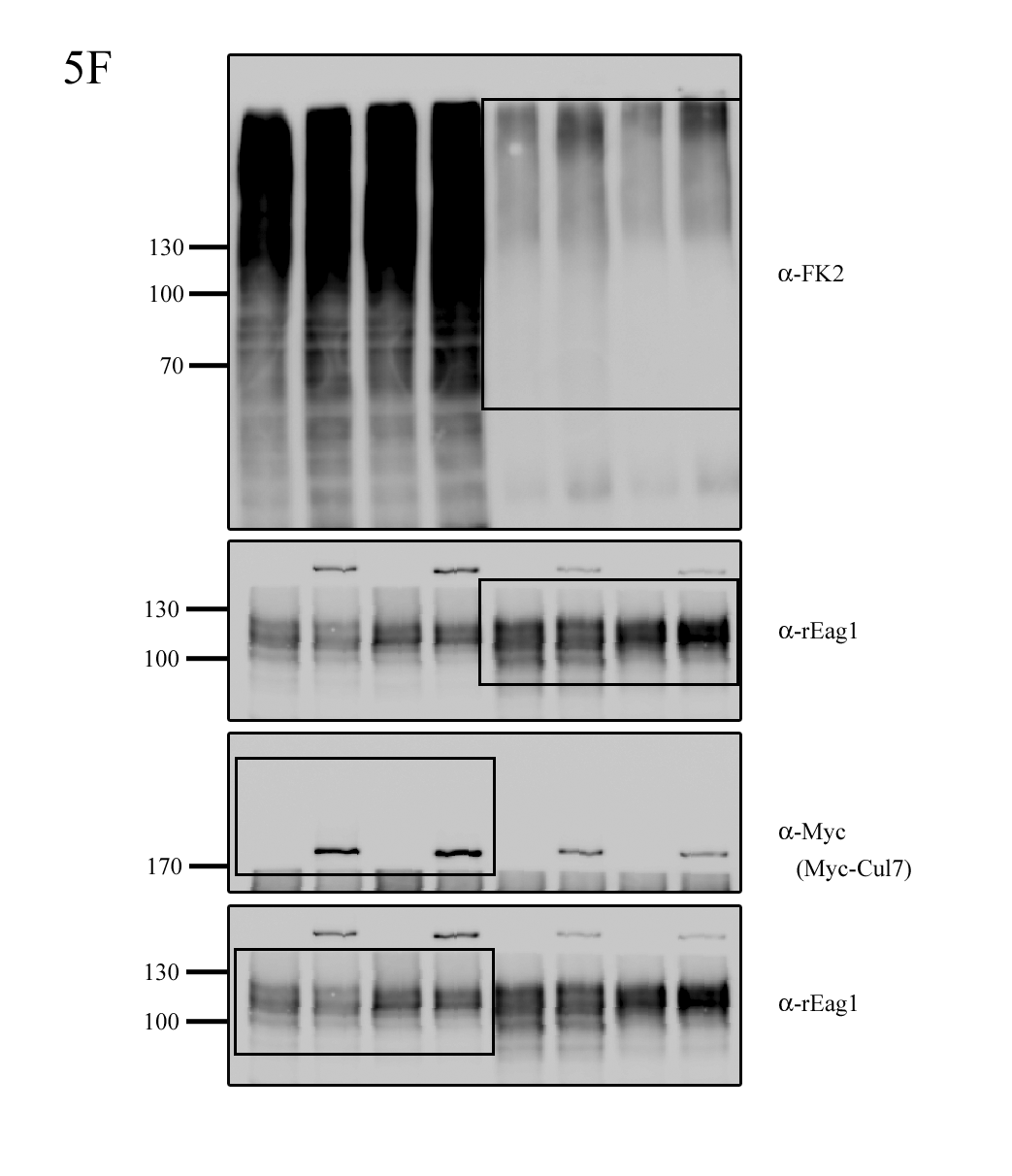


(related to Figure 6)


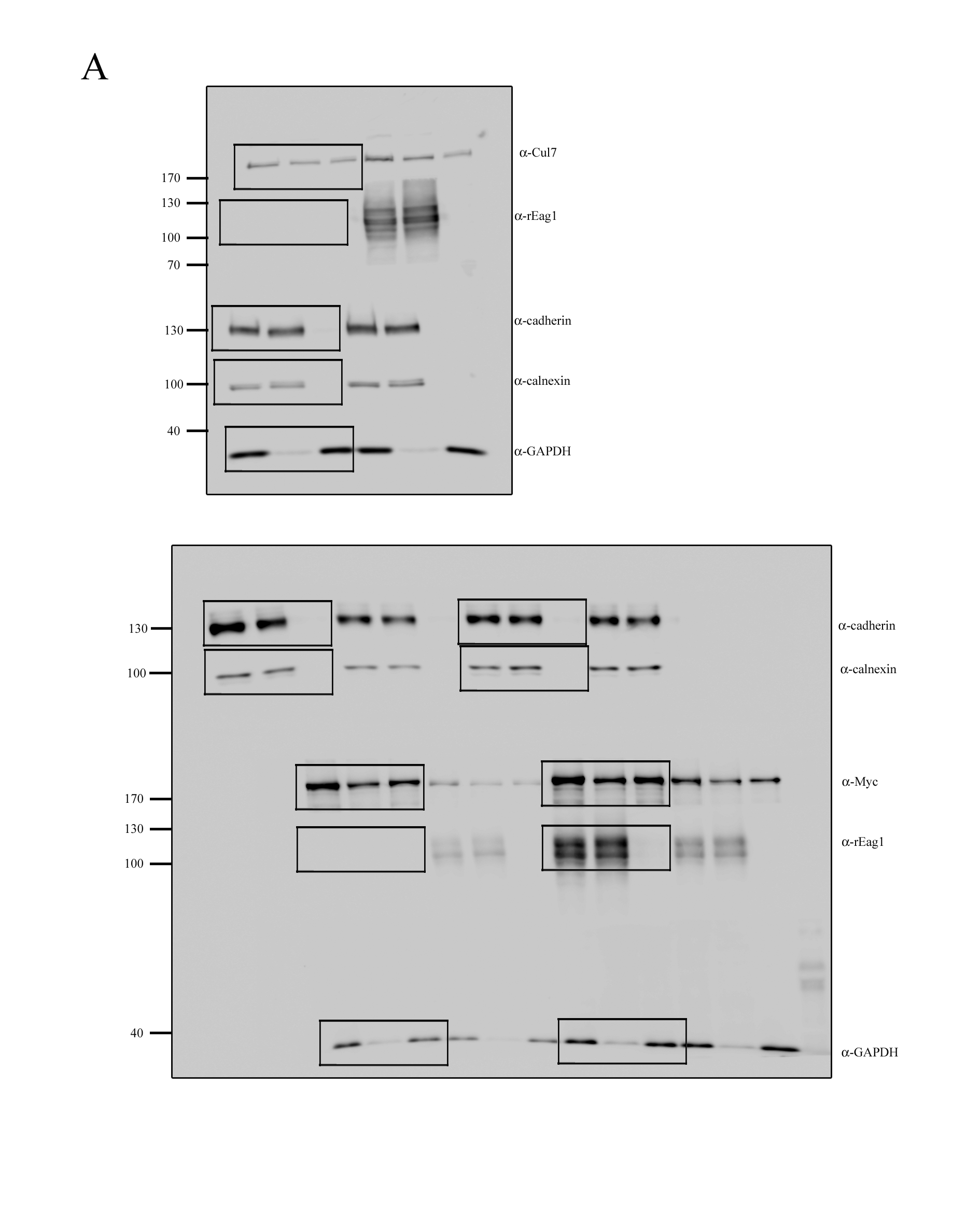


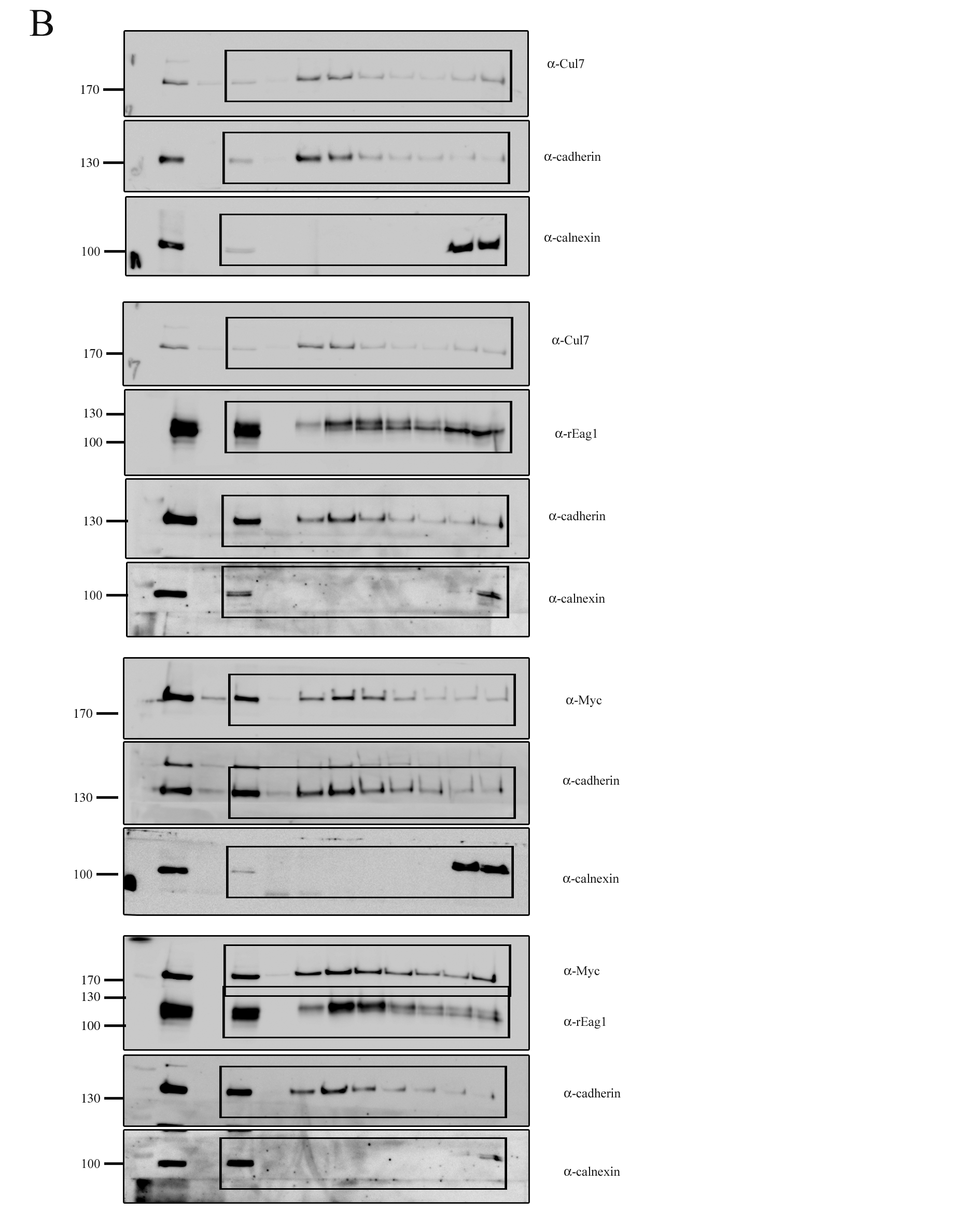


(related to Figure 8)


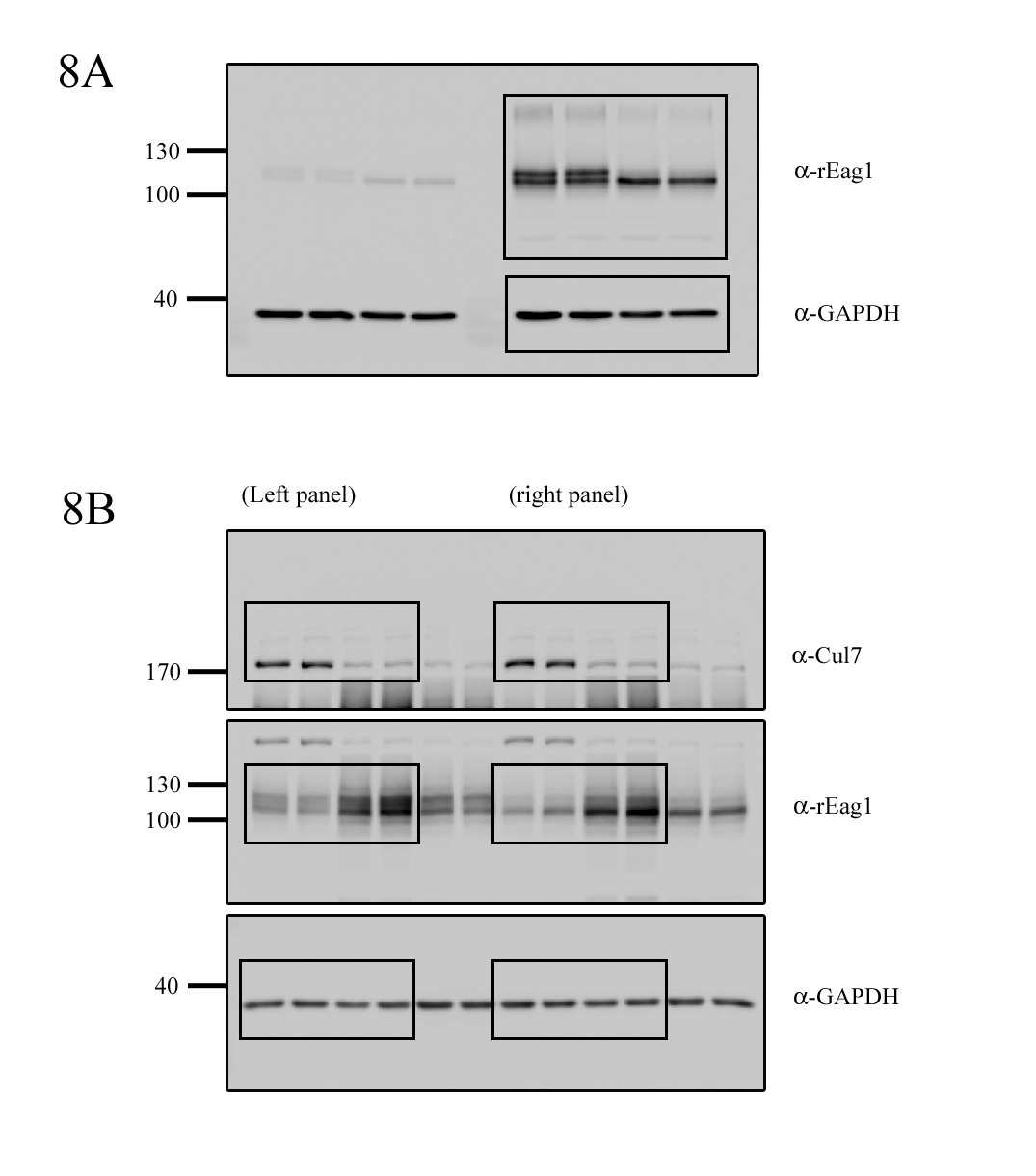

Supplement: Supplementary Information [file srep40825-s1.doc]
